# Quaternary climate change drives allo-peripatric speciation and refugial divergence in the *Dysosma versipellis-pleiantha* complex from different forest types in China

**DOI:** 10.1038/srep40261

**Published:** 2017-01-11

**Authors:** Yi-Han Wang, Hans Peter Comes, Ya-Nan Cao, Rui Guo, Yun-Rui Mao, Ying-Xiong Qiu

**Affiliations:** 1 Key Laboratory of Conservation Biology for Endangered Wildlife of the Ministry of Education, and Laboratory of Systematic & Evolutionary Botany and Biodiversity, College of Life Sciences, Zhejiang University, Hangzhou 310058, China; 2 Department of Ecology and Evolution, Salzburg University, A-5020 Salzburg, Austria

## Abstract

Subtropical China harbours the world’s most diverse temperate flora, but little is known about the roles of geographical and eco-climatic factors underlying the region’s exceptionally high levels of species diversity and endemism. Here we address this key question by investigating the spatio-temporal and ecological processes of divergence within the *Dysosma versipellis-pleiantha* species complex, endemic to subtropical China. Our cpDNA phylogeny showed that this monophyletic group of understory herbs is derived from a Late Pliocene ancestor of the Qinghai-Tibetan Plateau (QTP)/Southwest China. Genetic and ENM data in conjunction with niche differentiation analyses support that the early divergence of *D. versipellis* and *D. pleiantha* proceeded through allo-peripatric speciation, possibly triggered by Early Pleistocene climate change, while subsequent climate-induced cycles of range contractions/expansions enhanced the eco-geographical isolation of both taxa. Furthermore, modelling of population-genetic data indicated that major lineage divergences within *D. versipellis* likely resulted from long-term allopatric population isolation in multiple localized refugia over the last glacial/interglacial periods, and which in turn fostered endemic species formation (*D. difformis, D. majoensis*) from within *D. versipellis* in Southwest China. These findings point to an overriding role of Quaternary climate change in triggering essentially allopatric (incipient) speciation in this group of forest-restricted plant species in subtropical China.

Subtropical (Central/South/East) China (*c.* 22°N–30/33°N), the focal region of the present study, harbours the world’s most diverse temperate flora^1^, as mainly found in lower-elevation warm-temperate evergreen (WTE) vs. montane warm-temperate deciduous (WTD) forest (*c*. 300–800 m vs. *c*. 800–2000 m)^2^,^3^. However, little is known about the times and processes of diversification underlying the region’s exceptionally high species diversity and endemism. Recent phylogeographic studies of various plant species (e.g. trees, shrubs, herbs) representing either forest biome have provided evidence for long-term allopatric population isolation (viz. vicariance) across multiple localized refugia (e.g. WTD^4^,^5^,^6^,^7^,^8^,^9^; WTE^10^,^11^,^12^,^13^,^14^,^15^). For some of them (e.g. *Emmenopterys henryi*^8^), intraspecific genetic divergence has also been demonstrated to partly result from local adaptation to different climatic environments. However, as these recent efforts have all focussed on single species, it remains unclear to what extent geographical and/or eco-climatic factors have driven speciation among closely related plant taxa that vary in their geographic distributions and/or forest biome affinities across subtropical China.

Here we examine the evolutionary history of four closely related species of the ‘*Dysosma versipellis-pleiantha*’ complex (*sensu*^16^). Our preliminary analyses of nuclear ribosomal (nr) and chloroplast (cp) DNA^17^ placed this species complex as sister group to the rest of the genus, comprised of three high-elevation species of cool-temperate/subalpine forest (*c.* 1700–3900 m) from Yunnan (Southwest China) and the south-eastern slopes of the Qinghai-Tibetan Plateau (QTP); however, these analyses failed to further resolve relationships within the species complex. Notably, its four recognized species differ in their degree of range overlap and/or type of forest habitat across subtropical China (Fig. 1a,b)^16^,^18^. Two of them, *D. versipellis* (Hance) M. Cheng ex Ying and *D. pleiantha* (Hance) Woodson, form a parapatric species pair with abutting ranges in East China; the former occurs in isolated stands of montane WTD forest [*c.* 800–2000 (2700) m] throughout Central-East China, whereas *D. pleiantha* is associated with WTE forest habitats in the hilly/coastal areas of East China (300–800 m) and the mountains of Taiwan (1000–2500 m). By contrast, *D. difformis* (Hemsley & E.H. Wilson) T.H. Wang ex T.S. Ying and *D. majoensis* (Gagnepain) M. Hiroe are rare endemics with proximate but non-overlapping ranges in the WTD forests of Central-Southwest China, where both are broadly sympatric (but usually not locally admixed) with *D. versipellis* (Fig. 1b). Despite clear taxonomic arguments for the recognition of four species within the complex^16^,^18^, the possibility of hybridization has long been suspected from morphological and/or distributional considerations, e.g. especially between *D. versipellis* and both *D. pleiantha* and the subalpine *D. delavayi* (Franchet) Hu^16^.

In this study, we combined phylogeographic analyses using both nuclear and cpDNA data with cpDNA-based phylogenetic and dating approaches at the genus level, as well as Approximate Bayesian Computation (ABC), (palaeo-)climatic data and niche identity tests, to elucidate the spatial-temporal and ecological processes of divergence within the species complex that eco-geographically varies across the two major forest biomes of subtropical China. The specific aims of this study were: (1) to evaluate the phylogenetic relationships and divergence times of the four focal species and their intraspecific lineages; (2) to compare patterns of genetic diversity and structure among these four species; and (3) to infer the phylogeographic and demographic history of the species complex, including levels of admixture and/or hybridization.

## Results

### Chloroplast hapotype diversity and population structure

**Figure 1:**
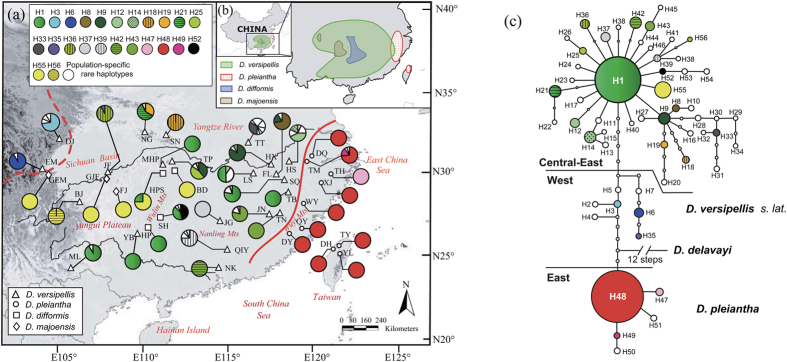
(**a**) Geographic distribution of the 56 chloroplast (cp) DNA (*trn*L‒*trn*F, *trn*L‒*ndh*J, *trn*S‒*trn*fM) haplotypes (H1–H56) detected in the *Dysosma versipellis-pleiantha* complex, created in illustrator v15.0 (http://www.adobe.com/products/illustrator.html) (see Supplementary Table S2 for population codes). Red dashed and solid lines denote the major western vs. central-east split within *D. versipellis s. lat.* (i.e. *D. versipellis, D. difformis, D. majoensis*) and the divergence between the latter and *D. pleiantha* further east, respectively, as identified by phylogenetic tree and network analyses. (**b**) Geographic ranges of *D. versipellis, D. pleiantha, D. difformis*, and *D. majoensis*^18^, created in illustrator. The base map was drawn using ArcGis v.9.3 (ESRI, Redlands, CA, USA). (**c**) Ninety-five percent statistical parsimony network of the 56 cpDNA haplotypes identified in the species complex generated in tsc v1.21 (http://darwin.uvigo.es/software/tcs.html). The small open circles represent missing haplotypes. The size of circles corresponds to the haplotype frequency.

The concatenated cpDNA sequences (*trn*L–*trn*F, *trn*L–*ndh*J, *trn*S–*trn*fM), surveyed across 452 individuals of the *D. versipellis-pleiantha* complex plus 10 accessions representing the five outgroup species, were aligned with a total length of 2604 bp, including 37 substitutions and 20 indels (1–23 bp). Within the species complex, these polymorphisms identified 56 haplotypes (Supplementary Table S1). Of those, 46 were specific to *D. versipellis* (H2–H46, H56), five to *D. pleiantha* (H47–H51) and three to *D. difformis* (H52–H54), while *D. majoensis* was fixed for H55 (Fig. 1a). H55 was shared between *D. difformis* and *D. majoensis*, and H1, the most widespread and common haplotype of *D. versipellis*, was also found in *D. difformis*. For the species complex as a whole, the data revealed high levels of haplotype diversity (*h*_T_ = 0.929) and nucleotide diversity (*π*_T_ = 3.58 × 10^−3^). The widespread *D. versipellis* had on average only slightly higher levels of within-population diversity (*h*_S_ = 0.300; *π*_S_ = 0.24 × 10^−3^) than the geographically more restricted *D. difformis* (*h*_S_ = 0.289; *π*_S_ = 0.14 × 10^−3^), whereas *D. pleiantha* was almost fixed for H48 (Supplementary Table S2; Fig. 1a). Hierarchical AMOVA apportioned 62% of the total cpDNA variance among the four species, whereas separate, non-hierarchical analyses revealed stronger population genetic structure in both *D. versipellis* (*Φ*_ST_ = 0.80) and *D. difformis* (*Φ*_ST_ = 0.63) compared to *D. pleiantha* (*Φ*_ST_ = 0.52) (Table 1). A significant cpDNA phylogeographic structure was indicated for the whole species complex (*N*_ST_ = 0.866 > *G*_ST_ = 0.790), *D. versipellis* (0.818 vs. 0.611) and *D. difformis* (0.865 vs. 0.728) (all *P* < 0.05), but not for *D. pleiantha* (0.204 vs. 0.161, *P* > 0.05).

### Phylogenetic haplotype relationships and molecular dating

**Figure 2:**
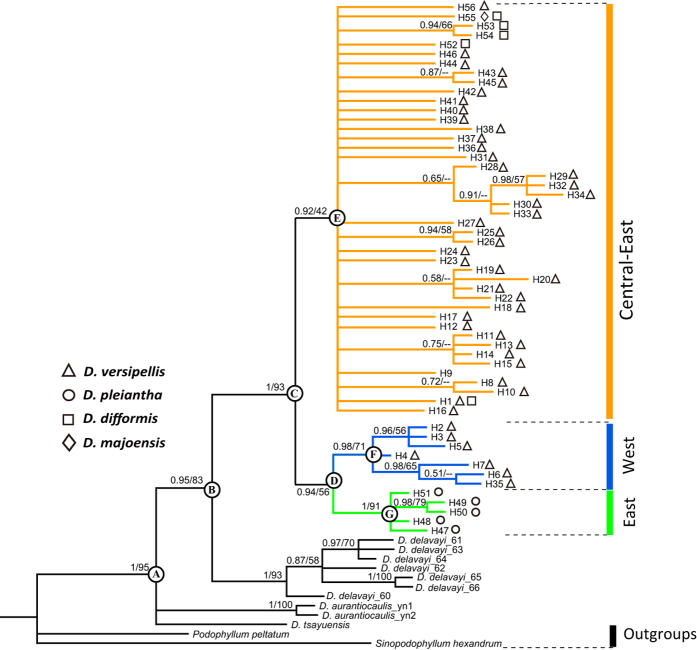
Bayesian inference (BI) phylogeny of the *Dysosma versipellis-pleiantha* complex based on cpDNA (*trn*L‒*trn*F, *trn*L‒*ndh*J, *trn*S‒*trn*fM) sequences with the remaining *Dysosma* species treated as part of the ingroup and *Podophyllum peltatum* and *Sinopodophyllum hexandrum* as outgroups. Posterior probabilities (PP > 0.50) and maximum likelihood (ML) bootstrap values (>50%) are sequentially indicated above the branches. Colored branches identify major haplotype lineages (west, central-east, east) within the species complex. Symbols following the haplotype numbers indicate the species bearing this haplotype. Nodes of interest are marked as A–G, while the corresponding beast-derived age estimates (including their 95% HPD intervals) are shown in Supplementary Table S3.

The cpDNA tree topologies obtained from Bayesian inference (BI) (Fig. 2) and maximum likelihood (ML, not shown) supported the monophyly of both *Dysosma* (PP = 1, ML = 95%) and the *D. versipellis-pleiantha* complex (PP = 1, ML = 93%), and resolved *D. delavayi* as most likely sister to the latter group (PP = 0.95, ML = 83%), while basal relationships between *D. aurantiocaulis* and *D. tsayuensis* remained unresolved. Within the species complex, three geographically structured lineages were resolved: (i) a well-supported, predominant ‘western’ lineage (PP = 0.98, ML = 71%), comprising all haplotypes of *D. versipellis* from the eastern slopes of the QTP (pops. EM, DJ: H2–H7), plus a haplotype (H35) unique to population JF from the Sichuan Basin (Fig. 1a); (ii) a weakly supported ‘central-eastern’ lineage (PP = 0.92, ML = 42%), containing all the remaining haplotypes of *D. versipellis*, and those of *D. difformis* and *D. majoensis* from Central-Southwest China; and (iii) a strongly-supported ‘eastern’ lineage (PP = 1, ML = 91%), consisting of all *D. pleiantha* haplotypes (Fig. 2). Although the ‘western’ and ‘eastern’ lineages appeared to be sisters, this relationship received very low support (PP = 0.94, ML = 56%). Individual mean age estimates for relevant nodes and their 95% highest posterior densities (HPDs) are provided for the different coalescent-type models in Supplementary Table S3. Accordingly, the *D. versipellis*-*pleiantha* complex likely originated in the Late Pliocene at *c.* 3.33 million yr ago (Ma) (constant size model) vs. *c.* 2.66 Ma (expansion growth model) (node ‘B’), and started to diversify in the Early Pleistocene (*c.* 2.16 vs 1.66 Ma) (node ‘C’), whereas the crown ages of the main lineages fell into the early-to-mid Pleistocene (central-east: *c.* 1.36 vs. 1.02 Ma, node ‘E’; west: *c.* 0.91 vs. 0.70 Ma, node ‘F’; east/*D. pleiantha*: *c.* 0.59 vs. 0.52 Ma, node ‘G’) (Supplementary Table S3).

In the rooted tcs network (Fig. 1c), all haplotypes of *D. pleiantha* were recovered as sister clade to the remainder, separated by five mutational steps. Within this latter clade (hereafter named ‘*D. versipellis s. lat.*’), haplotypes of the ‘western’ vs. ‘central-eastern’ lineages formed interior (ancestral) vs. tip (derived) subclades, again, separated by five steps. Notably, the low frequency haplotypes of both *D. difformis* (H52–H54) and *D. majoensis* (H55) appeared to be mutational derivatives of the most common and widespread haplotype of *D. versipellis* (H1) as part of the ‘central-east’ subclade.

### Demographic history

The mismatch distribution for cpDNA haplotypes of the ‘western’ lineage of *D. versipellis s. lat.* was bimodal, and thus differed strongly from that predicted under the spatial and demographic expansion models (Supplementary Fig. S1). This difference was also registered by non-significant values of both Fu’s *F*_S_ and Tajima’s *D* (Table 2). By contrast, both parameter values were significantly negative for the ‘central-east’ lineage of *D. versipellis s. lat.*, indicative of population expansion, and the same applied to *D. pleiantha* in terms of *F*_S_ (Table 2). Moreover, for either lineage, the MDAs gave unimodal graphs as well as non-significant *SSD* and *H*_*Rag*_ values under both the spatial and demographic models (Supplementary Fig. S1; Table 2). Based on the corresponding τ values and the assumed substitution rate of 1.3 × 10^−9^ s/s/y, we calculated a mid-Pleistocene expansion time for the ‘central-east’ lineage, regardless of the model used [demographic: *c*. 0.48 Ma (95% CI: 0.21–0.69 Ma); spatial: *c*. 0.36 (0.20–0.61) Ma]. For *D. pleiantha*, we obtained a similar timing under the demographic model [*c*. 0.43 (0.07–0.43) Ma], whereas the spatial model was more indicative of expansion during the Late Pleistocene/Holocene [*c*. 0.04 (0.0001–0.15) Ma].

### EST-SSR diversity, population structure and migration/gene flow

A total of 138 alleles were identified over the 15 EST-SSR loci among the 577 individuals (40 populations) surveyed. For each locus, the frequency of null alleles detected by freena was lower than the threshold (ν = 0.15) across all analyzed populations. There was no evidence for a systematic departure from HWE at particular loci, or a significant LD detectable among loci (data not shown). Using different outlier detection methods (see Supplementary Table S4), congruent evidence was found at each of three loci for either positive (EDV-46/52/119) or balancing selection (EDV-60/67/82).

At the species level, measures of genetic diversity derived from all 15 loci were highest in *D. versipellis* (e.g. *H*_E_ = 0.618), followed by *D. difformis* (*H*_E_ = 0.610), *D. pleiantha* (*H*_E_ = 0.473), and *D. majoensis* (*H*_E_ = 0.447; Supplementary Table S1). In the hierarchical AMOVA, 36.53% of the total EST-SSR variation was distributed among the four species (*R*_CT_ = 0.365), 34.54% was explained by variation among populations within species (*R*_SC_ = 0.544), and 28.93% was apportioned within populations (*R*_ST_ = 0.711; Table 1). Non-hierarchical analyses revealed stronger population genetic structure in *D. majoensis* (*R*_ST_ = 0.72) and *D. versipellis* (*R*_ST_ = 0.62), compared to *D. difformis* (*R*_ST_ = 0.38) and *D. pleiantha* (*R*_ST_ = 0.29) (Table 1).

**Figure 3:**
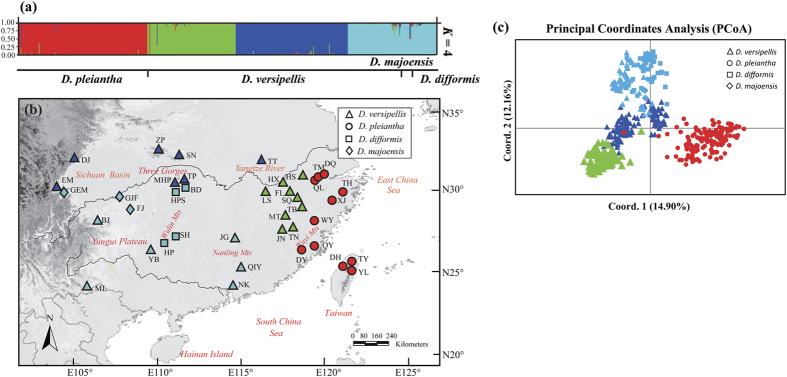
(**a**) Histogram of the assignment test for 40 populations (577 individuals) of the *Dysosma versipellis-pleiantha* complex based on genetic variation at 15 EST-SSR loci using structure v2.3.4 (http://pritchardlab.stanford.edu/structure.html). Each vertical bar represents one individual and its probability of membership for each of the *K* = 4 clusters. (**b**) Geographic distribution of the four structure clusters within and among populations of the species complex created in illustrator v15.0 (http://www.adobe.com/products/illustrator.html). The symbol next to each sampling locality identifies the respective *Dysosma* species, while the filled color represents the cluster assigned to that population (population codes are identified in Supplementary Table S2). The base map was drawn using ArcGis v.9.3 (ESRI, Redlands, CA, USA). (**c**) Principal coordinates analysis (PCoA) of the 577 individuals from the four species of the complex based on EST-SSR variation (15 loci) using arlequin v3.5 (http://cmpg.unibe.ch/software/arlequin35/).

In the structure analysis of the entire species complex using all 15 loci, ln *P*(D) progressively increased with *K*, whereas the ad hoc statistic Δ*K* showed the highest likelihood at *K* = 4 (Supplementary Fig. S2). Based on the latter model, all samples of *D. pleiantha* formed a distinct cluster (red in Fig. 3a,b), while those of *D. versipellis* segregated into three regional clusters, with populations originating from (i) both the west and the north (dark blue); (ii) the south (light blue); and (iii) the east (green). All samples of *D. difformis* and *D. majoensis* were assigned to the southern cluster of *D. versipellis* (Fig. 3a,b). Similarly, in the corresponding PCoA, the first axis distinguished individuals of *D. pleiantha* from all others, whereas individuals of *D. difformis* and *D. majoensis* broadly overlapped with the southern cluster of *D. versipellis* in genetic space (Fig. 3c). After removing the six outlier loci, Δ*K* favoured a model with *K* = 3. Running structure and using *K* = 3, *D. pleiantha* and eastern populations of *D. versipelli*s were assigned to two disctinct gene pools, whereas the reminder made up one single gene pool (see Supplementary Fig. S3). However, with *K* = 4, results were consistent with the clustering patterns inferred using all 15 loci (Supplementary Fig. S3).

The migrate analysis using all 15 loci revealed all pairwise estimates of migration/gene flow (*M*) among *D. pleiantha, D. difformis, D. majoensis* and the three regional clusters of *D. versipellis* to be low, ranging from 0.351 (*D. difformis* to west-northern cluster of *D. versipellis*) to 2.160 (eastern to west-northern cluster of *D. versipellis*) (Table 3). Basically the same results were obtained after removing the outlier loci (Table 3). Overall, these structure, PCoA and migrate analyses indicate only relatively minor effects of outlier loci on the overall population structure and gene flow of this species complex.

### ABC-based inferences of species/cluster divergence

In the diyabc analysis of the entire species complex based on the nine neutral EST-SSR loci, the highest posterior probabilities derived from the direct estimate approach were found for scenarios 1 (0.31, 95% CI: 0.00–0.71) and 4 (0.29, 0.00–0.69), whereas the values derived from the logistic regression were higher for the former scenario with no overlap of confidence intervals [0.46 (0.45–0.47) vs. 0.27 (0.26–0.28)] (Supplementary Fig. S4). In consequence, we favoured scenario 1, according to which the three regional structure clusters of *D. versipellis s. lat.* (west-north, east, south) diverged simultanously from a common ancestor, rather than any alternative hypothesis of ‘ordered’ relationships among these groups (Supplementary Fig. S5a, Table 4). The diyabc analysis further estimated that this simultaneous diversification of *D. versipellis s. lat.* occurred during the mid-Pleistocene (*c.* 0.59 Ma, 95% CI: 0.15–1.29 Ma; *t*_2_ in Supplementary Fig. S5a), while its divergence from *D. pleiantha* was dated to the Early Pleistocene [*c*. 0.92 (0.25–2.48) Ma; *t*_1_]. This latter timing broadly concurs with the beast-derived crown ages of the entire species complex estimated from cpDNA (2.25 and 1.81 Ma; see above).

In the analysis of the southern cluster (Supplementary Fig. S5b; Table 4), scenario 1 provided the best fit to the EST-SSR data [i.e. simultanous divergence of *D. versipellis* (south), *D. difformis, D. majoensis*], with posterior probability values being much higher than for the other three scenarios, regardless of the method used (direct estimate: 0.38, 0.18–0.47; logistic regression: 0.53, 0.52–0.54) (Supplemetntary Fig. S4). The time of diversification of this southern cluster was dated to the mid-Pleistocene [*c.* 0.43 (0.07–1.47) Ma; *t*_4_ in Supplementary Fig. S5b].

### Ecological niche modelling and niche identity tests

**Figure 4:**
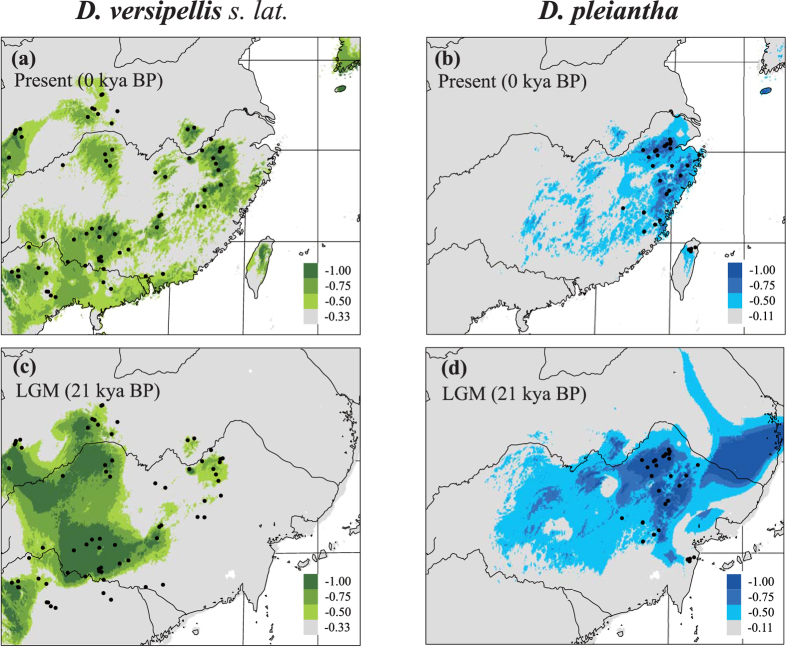
Predicted distributions of *D. versipellis s. lat.* (including *D. versipellis, D. difformis* and *D. majoensis*), and *D. pleiantha* under (**a**,**b**) current climate conditions (1950–2000) and (**c**,**d**) the Last Glacial Maximum (LGM; 21 kya BP) based on ecological niche modelling using maxent v3.3.1 (http://www.cs.princeton.edu/~schapire/maxent/). Maps were generated using ArcGis v9.3 (ESRI, Redlands, CA, USA).

The maxent models for *D. versipellis s. lat.* (including *D. difformis/D. majoensis*) and *D. pleiantha* had high predictive power and did not overfit the presence data (AUC values = 0.906 ± 0.118 and 0.972 ± 0.041, respectively). The current potential ranges of either group (defined as modelled suitability ≥0.75; Fig. 4a,b) were largely consistent with their actual distributions, except for predicted but unsupported occurrences of *D. versipellis s. lat.* in the far east of China, Taiwan, and South Korea (Fig. 4a). During the LGM (*c.* 21,000 yr BP), *D. versipellis s. lat.* experienced a drastic restriction of its current range in Central-East China (with only scattered areas persisting along the middle-Yangtze River), whereas suitable habitat apparently increased around the Sichuan Basin and areas further south (e.g. Southwest China, Yungui Plateau) (Fig. 4c). By contrast, for *D. pleiantha*, the LGM modelling predicted a more contiguous than currently scattered distribution in East China, extending southward into Taiwan (then connected with Mainland China), plus a higher suitability of areas both west- and eastward, i.e. south of the lower/middle Yangtze and on the then exposed East China Sea basin, respectively (Fig. 4d).

The results of the niche identity tests supported the existence of niche differentiation between the WTD forest-dweller *D. versipellis s. lat.* and its WTE forest counterpart *D. pleiantha*. Enmtools showed that the observed similarity values for *I* and *D* (0.818 and 0.582, respectively) were both significantly lower (*P* < 0.01) than the corresponding values expected from the pseudoreplicated datasets in the paired analyses (Supplementary Fig. S6).

## Discussion

Our cpDNA phylogeny (Fig. 2) confirms major relationships within *Dysosma* as hypothesized previously on the basis of cpDNA/nrDNA data^17^. In both analyses, the *D. versipellis-pleiantha* complex forms a strongly supported monophyletic group, which, in the present analysis, emerges from a paraphyletic and likewise supported grade of the remainder of the genus, comprised of *D. delavayi* (as sister to the complex), *D. aurantiocaulis*, and *D. tsayuensis*. Even though the species complex and the three latter, high-elevation species are not reciprocally monophyletic, they exhibit different morphologies^16^,^18^ and eco-geographies: while the former group is native to the lowland WTE or montane WTD forests of subtropical China, the latter is endemic to the cool-temperate/subalpine forests of Yunnan and the south-eastern QTP region. Given that the closest relative of *Dysosma, Sinopodophyllum hexandrum*, is native to the Himalayan regions^16^,^17^,^19^, we therefore assume that the most recent common ancestor (MRCA) of the *D. versipellis-pleiantha* complex originated in the high-altitude habitats of the QTP region/Southwest China, and then expanded its geographic range across subtropical China. In general, such a niche shift from high-to-lower elevation habitats is thought to be rare^20^, but is also known from other taxa in our study area (e.g. *Myricaria*^21^) or elsewhere (e.g. East African *Dendrosenecio*^22^; neotropical *Hypericum*^23^). The divergence of the *D. versipellis-pleiantha* complex from its closest high-altitude relative (*D. delavayi*) most likely occurred during the Late Pliocene (*c.* 3.33/2.66 Ma; Fig. 2), hence postdating a shift in East Asian climate toward drier conditions and a decrease in summer monsoon rainfall (*c.* 4–3 Ma^19^,^24^,^25^). These processes, in turn, may have reduced forest habitats^26^, which could have triggered the isolation of the lowland forms from highland forms.

Within the *D. versipellis-pleiantha* complex, the cpDNA phylogeny (Fig. 2) identified three geographic clades, albeit with different support: a well-supported western clade of *D. versipellis* (mainly distributed east of the QTP), a weakly supported ‘central-eastern’ clade (distributed in Southwest-Central-East China, and comprised of *D. versipellis* + *D. difformis* + *D. majoensis*), and a strongly supported eastern clade corresponding to *D. pleiantha*. By contrast, what emerges from our rooted cpDNA haplotype network (Fig. 1c) is striking evidence that *D. pleiantha* is sister to the remainder of the species complex (*D. versipellis s. lat.*). This inference of eco-geographically driven, and possibly adaptive divergence is further strengthened by our niche modeling, which suggests that these taxa occupy significantly different climatic environments (Supplementary Fig. S6). Moreover, in the cpDNA network, the central-eastern clade of *D. versipellis s. lat.* is placed in a derived (tip) position relative to the western (interior) one, and thus most distantly related to *D. pleiantha* (Fig. 1c). Hence, the present-day abutting ranges of *D. versipellis* and *D. pleiantha* in East China are secondary, rather than reflecting primary divergence between adjacent populations of a previously homogeneous species, i.e. a parapatric mode of speciation. We therefore conclude that the main mechanism, which caused the Early Pleistocene [0.92 (0.25–2.48) Ma] separation of the MRCA of *D. versipellis s. lat.* from eastern (‘peripheral’) forms that later gave rise to *D. pleiantha*, was vicariant allo-peripatric divergence at the extreme edge of an ancestral species’ range, perhaps triggered by the onset of the Pleistocene climate oscillations (*c.* 2.4–1.8 Ma). In other words, the peripheral forms of *D. pleiantha* would have persisted in East China under deteriorating conditions but became isolated by the retracting range of adjacent populations. Notably, this scenario resembles the rarely recognized ‘centrifugal speciation model’ (CSM) of allo-peripatric speciation^27^,^28^,^29^, and is also supported by the ENM results for the LGM (see below).

Our cpDNA-based estimates of demographic history (Table 2) provided clear evidence for spatial expansions in both the central-eastern clade of *D. versipellis s. lat.* during the mid-Pleistocene [*c*. 0.36 (0.20–0.61) Ma], possibly coinciding with the end of China’s ‘Penultimate Interglacial Period’ (*c.* 0.33–0.46 Ma^30^,^31^), and in *D. pleiantha* during the Late Pleistocene/Holocene [*c*. 0.04 (0.0001–0.15) Ma]. Yet, apparently, these expansions induced no secondary contact, as both taxa are identified here as genetically distinct units in terms of cpDNA haplotype (Fig. 1a,c) and EST-SSR variation (Fig. 3). Moreover, the migrate analyses of EST-SSRs uncovered extremely low levels of post-divergence gene flow between *D. versipellis* and *D. pleiantha* (Table 3), suggesting that their propensity for hybridization has been overstated in the taxonomic literature^16^. Rather, we suspect that both taxa, and their associated forest biomes, largely remained isolated from each other throughout the glacial and inter-/postglacial periods of the Quaternary. In fact, our ENM results indicate that, during the LGM (and possibly earlier cold periods), *D. versipellis s. lat.* and *D. pleiantha* occupied distinct refugia in West/Southwest China and East China, respectively, with only little potential range overlap in Central-East China (Fig. 4c,d). That glacial periods caused the range of *D. versipellis s. lat.* to retract south-westward (i.e. around the Sichuan Basin, Yungui Plateau), and/or to become fragmented, is similar with findings in other WTD forest species from subtropical China (e.g. *Cercidiphyllum japonicum*^6^; *Kalopanax septemlobus*^5^; but see below). However, that East China (and areas around the mid-/lower Yangtze) offered potentially suitable climate conditions for *D. pleiantha* during the LGM contrasts with fossil-pollen data^3^,^32^, suggesting that WTE forest largely retracted to the tropical South (≤24°N). Instead, our ENM data suggest that this drought-sensitive herb^16^ benefitted from relative climatic stability in East China over the last glacial cycle(s), possibly due to warm and moist air masses of monsoons from the ocean^33^,^34^ and/or topographic heterogeneity (e.g. Huang/Tianmu Mts.) that buffered against regional climate change^35^. The almost complete fixation of cpDNA haplotype H48 in *D. pleiantha* (Fig. 1a), together with its homogenous EST-SSR gene pool (red in Fig. 3), precludes more detailed inferences about the glacial refugia and (re-)colonization routes of this species. Nonetheless, when combined with the MDA evidence, this near lack of genetic structure most likely results from a founder effect in the wake of a leading edge expansion during the Late Pleistocene/Holocene^6^. Assuming this expansion signals recovery from a bottleneck^36^,^37^, any such earlier reduction in population size in *D. pleiantha* could have further enhanced its geographic isolation from *D. versipellis s. lat.* Moreover, any climate amelioration should have likewise promoted the isolation of these taxa. This hypothesis is based on the expectation that inter-/postglacial periods generally fostered the displacement of WTD forest into higher elevations by the expansion of WTE forest at lower elevations, as supported by phylogeographic studies^31^,^38^ and palaeo-data^32^. In sum, these findings indicate that the divergence between *D. versipellis s. lat.* and *D. pleiantha* has been triggered and maintained by geographical and ecological-adaptive processes in response to Pleistocene climate change.

Our survey of cpDNA sequence variation at 31 locations of *D. versipellis s. lat.* revealed substantial population differentiation (*Φ*_ST_ = 0.84) and significant phylogeographic structure (*N*_ST_ = 0.818 > *G*_ST_ = 0.611; *P* < 0.05). These patterns largely result from the major phylogeographic break across the Sichuan Basin between ancestral ‘western’ and derived (‘founded’) central-eastern populations, as well as the marked differences within the latter (Fig. 1). These results are similar to those of two earlier studies in *D. versipellis* based on cpDNA^31^ and AFLPs^39^, which also registered a major west vs. central-east split in this species, despite more limited samples (10 and nine populations, respectively). By contrast, based on our EST-SSR data, structure identified three geographical clusters in *D. versipellis s. lat.* (west-north, south, east). Hence, all populations of the western cpDNA lineage (EM, DJ) were found to share the same gene pool with five northern populations (ZF, SN, MHP, TP, TT) of the central-eastern lineage (Fig. 3a,b). This failure of the EST-SSRs to register the phylogeographic cpDNA split is probably best explained by extended times for lineage sorting to occur at nuclear compared to cytoplasmic loci^40^ rather than contemporary pollen-mediated introgression from the north into the west. The latter appears less likely if one considers not only the limited pollen-dispersal capacity of *Dysosma*^16^,^39^ but also the migrate results (Table 3), indicating only low levels of gene flow between the three EST-SSR clusters of *D. versipellis s. lat.* The previous cpDNA analysis of *D. versipellis*^31^ dated the separation between the western and central-eastern lineages to the mid-Pleistocene, about 0.48 Ma (95% CI: 0.18–0.86 Ma). In the present study, ABC analyses revealed that the divergence of the three regional EST-SSR clusters of *D. versipellis s. lat.* likewise dates back to the mid-Pleistocene (*c.* 0.59 Ma, 95% CI: 0.15–1.29 Ma; *t*_2_ in Supplementary Fig. S5a, Table 4). Even though difficult to compare across studies, and despite their broad confidence intervals, both time estimates strongly suggest that major lineage divergences within *D. versipellis s. lat.* long pre-date the LGM. Moreover, the present cpDNA and EST-SSR data provide a congruent picture of the phylogeographic history of *D. versipellis s. lat.*, as both support the hypothesis that populations of this taxon survived the (Late) Pleistocene glacial maxima in multiple refugia in both West/Southwest China and Central-East China. In the former region, there were probably three refugia: one, as identified by cpDNA, at the eastern slopes of the QTP (comprising populations DJ, EM; Fig. 1a); and two others, as revealed by the EST-SSRs, extending south vs. north-east of the Sichuan Basin (light vs. dark blue clusters; Fig. 3b,c). In Central-East China, there was probably a major refuge located south of the mid-/lower Yangtze, as demonstrated by the nuclear cohesion of all eastern populations (green cluster; Fig. 3b,c), and possibly a more localized one further north in the Dabie Mts., as might be inferred from the high number of unique cpDNA haplotypes in the ‘north-cluster’ population TT (Fig. 1a, Supplementary Table S2). Notably, these latter two refugia are also predicted by the ENM of *D. versipellis s. lat.* for the LGM (Fig. 4c); on the other hand, the genetic data better serve to illustrate that West/Southwest China did not function as a single refugium for this WTD forest species during glacial periods but instead hosted different nuclear and/or cytoplasmic lineages in separate refugia. The occurrence of these ‘refugia-within-refugia’^41^ not only caused the allopatric divergence of these lineages but probably also fostered the recent speciation of *D. difformis and D. majoensis* from within *D. versipellis*.

*Dysosma difformis* and *D. majoensis* have proximate but non-overlapping eastern vs. western distributions along the southern fringe of the Sichuan Basin (Fig. 1a,b). These two narrow endemics share the same WTD forest habitat with *D. versipellis* (*s. str.*) and are sometimes difficult to distinguish from the latter based on morphology, as most of their diagnosable features relate to aspects of leaf lobing^16^,^18^. Our genetic data clearly suggest a close relationship among these three taxa, without traces of other *Dysosma* species (e.g. *D. delavayi*) as suggested previously for *D. majoensis*^16^. Rather, all cpDNA haplotypes unique to *D. difformis* (H52–H54; only population SH) and the single one fixed in *D. majoensis* (H55) are independent mutational derivatives of the most common and widespread haplotype of *D. versipellis* (H1; see Fig. 1a,c). Moreover, the EST-SSR data show that *D. difformis* and *D. majoensis*, despite sharing the same gene pool (light blue) with all southern populations of *D. versipellis* (Fig. 3), have likely exchanged only few migrants with each other and the latter species in the distant past (Table 3). Finally, the corresponding ABC analysis favoured a scenario of unresolved relationships, according to which *D. difformis, D. majoensis* and *D. versipellis* (southern cluster) evolved independently from a common ancestor, possibly during the mid-Pleistocene [*c.* 0.43 (0.07–1.47) Ma; *t*_4_ in Supplementary Fig. S5b]. Taken together, these data seem to support a scenario in which climate deterioration following China’s penultimate interglacial period (*c.* 0.33–0.46 Ma; see above) promoted the independent origin of *D. difformis* and *D. majoensis* from within *D. versipellis* through population isolation in two separate refugia along the southern Sichuan Basin. If so, the presence of cpDNA haplotypes H1 and H55 in *D. difformis* (Fig. 1a,c) most likely results from, respectively, incomplete lineage sorting (ILS; i.e. recent descent from *D. versipellis*) and past chloroplast introgression from *D. majoensis*, perhaps facilitated by massive expansion of suitable habitat in the area during the LGM (Fig. 4c). Clearly, the present data cannot fully disentangle these hypotheses of recent speciation, ILS and/or past admixture; however, they do suggest that the combined effects of these historical processes, rather than contemporary gene flow, are responsible for the limited evidence of genetic (cytoplasmic/nuclear) divergence of *D. difformis* and *D. majoensis*. These data, therefore, should not be interpreted to reject the hypothesis of geographic-reproductive isolation of these two endemics and their evolutionary distinctiveness as taxonomic units of interest^42^,^43^; rather, they underscore the mountain regions of Southwest China of central importance for (micro-)refugial allopatric speciation^31^,^44^.

In summary, the data presented here for the *D. versipellis*-*pleiantha* complex suggest an overriding role of Quaternary climate change in triggering essentially allopatric (incipient) speciation in this group of forest-restricted plant species in subtropical China.

## Materials and Methods

### Plant material and sampling design

The ‘*D. versipellis-pleiantha*’ complex comprises four closely related species (*D. pleiantha, D. versipellis, D. difformis* and *D. majoensis*) of the diploid (2*n* = 12) perennial understory herb genus *Dysosma* Woodson (Berberidaceae, Podophylloideae; *c.* 7 spp.). All four species have self-incompatible, dull/red-coloured, and insect (carrion fly/beetle) pollinated flowers that mature into purplish-red berries^16^,^39^,^45^,^46^, and, like all species of *Dysosma*, are able to produce fertile F_1_ hybrids^16^,^46^. In this study, we sampled a total of 618 individuals from 42 localities of *D. versipellis* (24), *D. pleiantha* (11), *D. difformis* (4), and *D. majoensis* (3) (Supplementary Table S2, Fig. 1). Together, this sampling covers most of the distribution range of this species complex. Voucher specimens representative of all populations sampled are stored at the Herbarium of Zhejiang University (HZU). Of these 42 populations, five populations were only surveyed for either cpDNA (pop. JF and NG) or EST-SSR loci (pop. MT, ZP and QL), given the small amount of leaf material available. Accordingly, a total of 577 individuals from 40 populations were employed for the EST-SSR analysis, whereas 452 individuals from 39 populations were also surveyed for cpDNA sequence variation (Supplementary Table S2). For the phylogenetic cpDNA analyses (see below), we also collected samples from the three remaining *Dysosma* species, i.e. *D. aurantiocaulis* (Handel-Mazzetti) Hu (1 individual), *D. delavayi* (6), and *D. tsayuensis* T.S. Ying (1), plus one individual each of the closely related *Sinopodophyllum hexandrum* (Royle) Ying and *Podophyllum peltatum* L.^19^,^47^.

### DNA extraction, cpDNA sequencing, and EST-SSR genotyping

Total genomic DNA was extracted from the silica-dried leaf materials using DNA Plantzol (Invitrogen) following the manufacturer’s instructions. For the phylogeographic and phylogenetic cpDNA analyses, we sequenced three non-coding intergenic spacer (IGS) regions, i.e. *trn*L–*trn*F, *trn*L–*ndh*J^48^ and *trn*S–*trn*fM^49^. Sequences were generated with an ABI 377XL DNA sequencer (Applied Biosystems), and edited, assembled, and aligned in geneious v7.1.7^50^.

Fifteen EST-SSR primer pairs, developed from the transcriptome of *D. versipellis* (termed ‘EDV’ primers), were employed for genotyping all 577 DNA samples. Ten of those primers (EDV-30, 37, 40, 46, 52, 53, 54, 59, 60, 67) were described in Guo *et al*.^51^ (GenBank accession numbers: KJ000290–KJ000299). Respective primers for the other five loci (EDV-81, 82, 102, 118, 119) were developed in this study (Supplementary Table S6). All loci were proved to be consistently variable and transferable among the four species of the *D. versipellis-pleiantha* complex after a pilot study. Amplification of the EST-SSR loci followed the protocol of Guo *et al*.^51^. Fluorescently labelled PCR products were supplemented with the internal size standard GS-500 and separated on a MegaBACE 1000 (GE Healthcare Biosciences, Sunnyvale, CA, USA). Alleles were scored manually in genemarker v2.20 (SoftGenetics, State College, PA, USA).

### Chloroplast hapotype diversity and population structure

The number of haplotypes, haplotype diversity (*h*), and nucleotide diversity (*π*) were calculated at the levels of populations (*h*_S_, *π*_S_) and species (*h*_T_, *π*_T_) using dnasp v5.0^52^. Non-hierarchical and hierarchical analyses of molecular variance (AMOVAs) were carried out in arlequin v3.5^53^ with significance of *Φ*-statistics tested by 1000 non-parametric random permutations. The presence of phylogeographic structure was tested across all populations of the species complex and for each species and/or lineage separately using permut v1.0^54^. All haplotype sequences identified in the present study were deposited in GenBank with accession numbers (see Supplementary Table S7 for details).

### Phylogenetic haplotype relationships and molecular dating

Phylogenetic cpDNA haplotype trees of the species complex were constructed using maximum likelihood (ML) and Bayesian inference (BI) methods, with the remaining *Dysosma* species treated as part of the ingroup and *S. hexandrum* and *P. peltatum* as outgroup (see Supplementary Methods for details). For the phylogeographic cpDNA analyses, a haplotype network was constructed in tcs v1.21^55^ under the 95% statistical parsimony criterion with gaps (indels) coded as substitutions (A or T), and rooted with *D. delavayi*.

Divergence date analyses were conducted on the cpDNA data set in beast v1.80^56^,^57^ using the GTR + G substitution model (see Supplementary Methods for details) under the assumption of an uncorrelated lognormal relaxed clock^56^. As there are no known fossil records in Podophylloideae, we employed a substitution rate of 1.3 × 10^−9^ substitutions per site per year (s/s/y) as estimated for non-coding chloroplast regions of *D. versipellis*^31^. We implemented two coalescent-type models, assuming either a constant population size or population expansion. For each model, four independent Markov chain Monte Carlo (MCMC) runs of 50 million generations each were performed with sampling every 5000 generations, following a burn-in of the initial 10% cycles. We used TreeAnnotator v1.6.1^57^ to construct a maximum clade credibility tree.

### Demographic analyses

To infer the demographic history of major cpDNA lineages within the species complex, we tested the null hypotheses of a spatial expansion and a pure demographic expansion using mismatch distribution analysis (MDA) in arlequin. For two expanding lineages identified, the MDA-derived expansion parameter (τ) and its 95% confidence interval (CI) were converted to absolute estimates of time since expansion (see Supplementary Methods for more details). In addition, we used tests of selective neutrality [Tajima’s *D*^58^; Fu’s *F*_s_^59^] to infer potential population growth and expansion.

### Population structure and migration/gene flow inferred from EST-SSR markers

For the EST-SSR dataset, all 15 genotyped loci were checked for frequencies of null alleles, deviations from Hardy-Weinberg equilibrium (HWE) and linkage disequilibrium (LD) (see Supplementary Methods for details). For each locus, we also tested potential signatures of selection in lositan^60^, arlequin, and bayescan v2.1^61^. Following De Mita *et al*.^62^, we considered a locus to be under selection if at least two outlier tests were significant for that particular locus. Using fstat, the following diversity and inbreeding parameters were calculated for each population and across all 15 loci: *N*_A_, numbers of alleles; *R*_S_, allelic richness^63^; *H*_E_; and the average inbreeding coefficient (*F*_IS_). Hierarchical and non-hierarchical AMOVAs were carried out in arlequin using *R*-statistics and significance tests as described above for cpDNA.

Genetic subgroups of the species complex were identified by Bayesian analyses in structure v2.3.4^64^, using the admixture model and assuming independent allele frequencies among populations (see Supplementary Methods for details). Clustering analyses for the EST-SSRs were complemented with an individual-based principal coordinates analysis (PCoA) using software genalex v6.5^65^.

We also used coalescent-based methods implemented in migrate-n v3.6^66^ to obtain pairwise estimates of mutation-scaled migration rate (*M*) over *c.* 4*N*_e_ generations in the past^67^ between species and/or intraspecific regional clusters (only *D. versipellis*; see structure results). All analyses were carried out with and without outlier loci included to assess their effects on the inference of population structure and gene flow^68^. More software running details can be found in the Supplementary Materials.

### Testing for the times and orders of species/cluster divergence using ABC

We used ABC simulations in diyabc v2.0^69^,^70^ to gain further insights into the times and orders of divergence among clusters identified by structure based on the nine neutral EST-SSR loci. In a first analysis (‘a’), we tested the simultanous divergence of the three clusters of ‘*D. versipellis s. lat.*’ (i.e. west-north, east, south, with the latter including *D. difformis*/*D. majoensis*; see Results) from a common ancestor against three alternative models, reflecting all possible relationships among these clusters (see scenarios 1–4 in Supplementary Fig. S5a and Table S5). For each scenario, *D. pleiantha* was assumed as sister, based on the cpDNA haplotype network analysis (see Results). In a second analysis (‘b’), we tested four similar divergence order hypotheses between the taxonomic units of the southern cluster, i.e. *D. versipellis* (south), *D. difformis* and *D. majoensis* (scenarios 1–4 in Supplementary Fig. S5b and Table S5). Additional details in data simulation, scenario comparison, parameter estimation, etc. can be found in the Supplementary Materials.

### Ecological niche modelling and niche identity tests

Ecological niche modelling (ENM) was carried out in maxent v3.3.1^71^,^72^ to predict suitable past and present climate envelopes for, respectively, the WTD forest species (i.e. *D. versipellis s. lat.*, including *D. versipellis, D. difformis, D. majoensis*) and the WTE forest species *D. pleiantha*. Information on the geographic distribution of the three former species was based on the 31 populations included in this study, combined with 102 presence records obtained from the Chinese Virtual Herbarium (CVH) (www.cvh.org.cn) and the National Specimen Information Infrastructure of China (NSII) (www.nsii.org.cn). In addition, 44 records were available for *D. pleiantha*, i.e. 11 localities of this study plus 33 obtained from CVH and NSII.

Current distributions of the two sets of species were modelled using six bioclimatic data layers (annual mean temperature, annual precipitation, precipitation of wettest, driest, warmest and coldest quarter) available from the WorldClim database (http://www.worldclim.org)^73^ at 2.5 arc-min resolution for the present (1950–2000). This restricted bioclimatic dataset avoided including highly correlated variables, and thus prevented potential over-fitting^74^. This model was then projected onto the palaeoclimate dataset simulated by the community climate system model v3.0^75^ to infer the extent of suitable habitat during the Last Glacial Maximum (LGM; *c.* 21 kya BP). Model performance was evaluated using the area under the ‘Receiver Operating Characteristic (ROC) Curve’ (AUC) calculated by maxent. Values between 0.7 and 0.9 indicate good discrimination^76^.

We also performed niche identity tests in enmtools v1.4.3^77^ based on all the 19 BIOCLIM variables from the WorldClim dataset for testing the null hypothesis that the WTD vs. WTE forest species (as defined above) are occupying identical climatic environments (‘niches’). However, we did not further evaluate the niche similarity between *D. difformis* and *D. majoensis* because of the very low number of known occurrence records for either species. Niche overlap was quantified using the standardized Hellinger distance (*I*) and Schoener’s *D*^78^.

### Data Availability

cpDNA (trnL–trnF, trnF–ndhJ and trnS–trnfM) sequences obtained in this study are deposited in GenBank (see Supplementary Table S7 for accession numbers). Microsatellite genotypes and maxent input file have been archived with Dryad (doi: 10.5061/dryad.9d8k4).

## Additional Information

**How to cite this article**: Wang, Y.-H. *et al*. Quaternary climate change drives allo-peripatric speciation and refugial divergence in the *Dysosma versipellis-pleiantha* complex from different forest types in China. *Sci. Rep.* **7**, 40261; doi: 10.1038/srep40261 (2017).

**Publisher's note:** Springer Nature remains neutral with regard to jurisdictional claims in published maps and institutional affiliations.

## Supplementary Material

Supplementary Information

## Figures and Tables

**Table 1:** Results of analyses of molecular variance (AMOVAs) based on cpDNA haplotype data and EST microsatellite allele frequencies for species and populations of the *D. versipellis-pleiantha* complex.

Source of variation	cpDNA			EST-SSR (All 15 loci/nine neutral loci)		
	d.f.	Percentage of variation (%)	*Φ*-statistics	d.f.	Percentage of variation (%)	*R*-statistics
*D. versipellis-pleiantha* complex						
Among species	3	62.45	*Φ*_CT_ = 0.62	3/3	36.53/14.09	*R*_CT_ = 0.365/0.141
Among populations within species	34	29.97	*Φ*_SC_ = 0.80	36/36	34.54/46.65	*R*_SC_ = 0.544/0.543
Within populations	425	7.58	*Φ*_ST_ = 0.92	1114/1114	28.93/39.27	*R*_ST_ = 0.711/0.607
*D. versipellis-pleiantha* complex						
Among cpDNA lineages	2	74.91	*Φ*_CT_ = 0.75			
Among populations within lineages	34	18.67	*Φ*_SC_ = 0.74			
Within populations	425	6.42	*Φ*_ST_ = 0.94			
*Dysosma versipellis*						
Among populations	20	80.40	*Φ*_ST_ = 0.80	21/21	61.60/61.69	*R*_ST_ = 0.616/0.617
Within populations	269	19.60		679/679	38.40/38.30	
*Dysosma pleiantha*						
Among populations	9	51.77	*Φ*_ST_ = 0.52	10/10	28.97/24.16	*R*_ST_ = 0.290/0.242
Within populations	114	48.23		345/345	71.03/75.84	
*Dysosma difformis*						
Among populations	3	62.91	*Φ*_ST_ = 0.63	3/3	37.64/29.45	*R*_ST_ = 0.376/0.294
Within populations	31	37.09		65/65	62.35/70.55	
*Dysosma majoensis*						
Among populations				2/2	71.90/72.55	*R*_ST_ = 0.719/0.725
Within populations				25/25	28.10/27.45	

All levels of variation were significant. Note that *D. majoensis* was proved invariable at the cpDNA level.

**Table 2:** Mismatch distribution analysis (MDA) of the three cpDNA lineages of the *D. versipellis*-*pleiantha* complex (west, central-east: *D. versipellis s. lat*.; east: *D. pleiantha*) for pure demographic and spatial expansion models, tested with the sum of squared deviations (*SSD*) and Harpending’s^79^ raggedness index (*HRag*) in ****arlequin** **.

Model	Lineage	Parameter (τ)	Expansion time (*t*) in yr BP	*SSD*	*P*	*HRag*	*P*	Fu’s *F*_*S*_	*P*	Tajima’s *D*	*P*
Demographic expansion	West	4.910 (0.207‒8.426)	NC	0.1349	0.05	0.368	0.01	−0.19	0.47	0.5	0.71
	Central-east	3.350 (1.455‒4.800)	**482,750** (209,672‒691,702)	0.0005	0.92	0.015	0.87	−22.3	0.00	−1.59	0.02
	East	3.000 (0.512‒3.000)	**432,314** (73,782‒432,314)	0.0000	0.47	0.562	0.67	−3.06	0.02	−1.24	0.07
Spatial expansion	West	4.270 (1.791‒7.839)	NC	0.0985	0.08	0.368	0.25	−0.19	0.45	0.50	0.74
	Central-east	2.474 (1.402‒4.207)	**356,515** (202,034‒606,248)	0.0004	0.96	0.015	0.90	−22.3	0.00	−1.59	0.02
	East	0.255 (0.001‒1.035)	**36,747** (114‒149,148)	0.0000	0.55	0.562	0.66	−3.06	0.02	−1.24	0.09

See Supplementary Fig. S1 for illustrations of mismatch distributions. Also shown are results of selective neutrality tests (Fu’s *F*_S_, Tajima’s *D*). For the *F*_S_ test, *P* = 0.02 is considered to be significant at the α = 0.05 level^59^,^80^. NC, not calculated.

**Table 3:** Estimates of historical migration rate (*M*) and 95% confidence intervals (CI) (in parentheses) among species of the *D. versipellis-pleiantha* complex and regional clusters of *D. versipellis* using all 15 (in bold) and mere nine neutral EST-SSR loci.

	*D. pleiantha*	*D. versipellis* East (E)	*D. versipellis* West-north (WN)	*D. versipellis* South (S)	*D. majoensis*	*D. difformis*
*D. pleiantha*		**1.343 (1.165‒1.540)**	**1.887 (1.454‒2.125)**	**1.018 (0.862‒1.348)**	**0.457 (0.356‒0.575)**	**0.377 (0.286‒0.485)**
		2.617 (2.232‒2.958)	1.337 (1.078‒1.580)	0.844 (0.658‒1.044)	0.851 (0.656‒1.047)	0.071 (0.004‒0.262)
*D. versipellis*E	**1.887 (1.659‒2.136)**		**0.808 (0.586‒0.974)**	**0.626 (0.499‒0.773)**	**0.483 (0.341‒0.615)**	**0.351 (0.252‒0.465)**
	1.479 (1.211‒1.759)		1.196 (0.984‒1.471)	0.398 (0.279‒0.560)	0.625 (0.475‒0.804)	0.304 (0.134‒0.434)
*D. versipellis*WN	**1.531 (1.342‒1.737)**	**2.160 (1.936‒2.401)**		**1.044 (0.891‒1.213)**	**0.950 (0.804‒1.114)**	**0.443 (0.346‒0.601)**
	2.495 (2.127‒2.827)	1.828 (1.568‒2.162)		1.086 (0.836‒1.310)	0.578 (0.411‒0.745)	0.411(0.295‒0.559)
*D. versipellis*S	**0.572 (0.452‒0.710)**	**1.118 (0.947‒1.308)**	**1.126 (0.863‒1.319)**		**1.069 (0.903‒1.255)**	**1.027 (0.864‒1.210)**
	0.470 (0.334‒0.638)	1.050 (0.830‒1.445)	0.589 (0.434‒0.790)		1.581 (1.294‒1.877)	0.873 (0.683‒1.097)
*D. majoensis*	**0.174 (0.098‒0.286)**	**0.526 (0.388‒0.692)**	**0.625 (0.473‒0.808)**	**1.160 (0.944‒1.403)**		**1.042 (0.835‒1.279)**
	0.144 (0.076‒0.244)	0.772 (0.594‒0.983)	0.773 (0.508‒1.025)	0.246 (0.151‒0.391)		0.304 (0.197‒0.747)
*D. difformis*	**0.681 (0.525‒0.866)**	**0.550 (0.412‒0.717)**	**1.096 (0.897‒1.323)**	**1.347 (1.123‒1.603)**	**1.483 (1.247‒1.748)**	
	0.662 (0.474‒0.947)	0.669 (0.464‒0.898)	0.908 (0.408‒1.169)	1.376 (1.100‒1.695)	0.875 (0.620‒1.133)	

Directionality of gene flow is read from top species/clusters being the source populations, whereas units on the left are the recipient species/clusters.

**Table 4:** Descriptions of prior settings and median estimations of posterior distributions of parameters revealed by the diyabc modeling of the best-fitting scenarios for the diversification history of (a) the three regional EST-SSR **( **structure** )** clusters of *D. versipellis s. lat.* (west-north, east, south); and (b) the three taxonomic units of its ‘southern cluster’, i.e. *D. versipellis* (south), *D. difformis*, and *D. majoensis*.

Analysis/scenario no.	Parameter	Median	95% lower bound	95% upper bound
(a) Scenario 1				
	NA	137 000	11 300	727 000
	NDP	473 000	187 000	825 000
	NDV_(WN)_	593 000	257 000	899 000
	NDV_(E)_	316 000	106 000	714 000
	NDV_(S1)_	456 000	170 000	838 000
	*t*_1_ (in million years ago)	0.92	0.25	2.48
	*t*_2_ (in million years ago)	0.59	0.15	1.29
	*μ*	1.99 × 10^–6^	1.16 × 10^–6^	4.66 × 10^–6^
	*P*	0.38	0.14	0.80
(b) Scenario 1				
	NA	290 000	2 7500	860 000
	NDV_(S2)_	459 000	135 000	871 000
	NDD	471 000	139 000	880 000
	NDM	277 000	68 800	755 000
	*t*_4_ (in million years ago)	0.43	0.07	1.47
	*μ*	2.03 × 10^–6^	1.14 × 10^–6^	6.45 × 10^–6^
	*P*	0.29	0.12	0.74

See Materials and Methods, Supplementary Fig. S5, and Table S5 for details. In (a), NDP, NDV_(WN)_, NDV_(E)_, and NDV_(S1)_ denote the current population sizes of, respectively, *D. pleiantha, D. versipellis* west-north cluster, *D. versipellis* east cluster and *D. versipellis* south cluster (including *D. difformis* and *D. majoensis*). In (b), NDV_(S2)_, NDD, and NDM represent the current population sizes of, respectively, *D. versipellis* south cluster, *D. difformis* and *D. majoensis.* NA represents the population size of the ancestral lineage at the basal node. *μ* denotes the mutation rate (per generation per locus) for the EST-SSRs. *P* represents the proportion of multiple step mutations in the generalized stepwise model, GSM.
