# Supplementary material for: Quaternary climate change drives allo-peripatric speciation and refugial divergence in the Dysosma versipellis-pleiantha complex from different forest types in China
Source: Sci Rep. 2017 Jan 11;7:40261. doi: 10.1038/srep40261 (PMC5225488; doi:10.1038/srep40261)
Supplement: Supplementary Information [file srep40261-s1.pdf]

Supplementary Information

Quaternary climate change drives allo-peripatric speciation and refugial divergence in the *Dysosma versipellis-pleiantha* complex from different forest types in China

Yi-Han Wang[1], Hans Peter Comes[2], Ya-Nan Cao[1], Rui Guo[1], Yun-Rui Mao[1] & Ying-Xiong Qiu[1]*

[1] Key Laboratory of Conservation Biology for Endangered Wildlife of the Ministry of Education, and Laboratory of Systematic & Evolutionary Botany and Biodiversity, College of Life Sciences, Zhejiang University, Hangzhou 310058, China, [2]Department of Ecology and Evolution, Salzburg University, A-5020 Salzburg, Austria

*Corresponding author. Ying-Xiong Qiu. E-mail: qyxhero@zju.edu.cn

Supplementary Methods

Reconstruction of phylogenetic cpDNA haplotype trees

The best-fit substitution model employed was GTR+G as determined by Akaike Information Criteria (AIC) implemented in JMODELTEST (v1.1; Posada, 2008). The ML analysis was conducted using RAXML (v7.2.8; Stamatakis *et al.*, 2008) at the CIPRES SCIENCE GATEWAY (v3.1; Miller *et al.*, 2010). Nodal support was assessed using 1000 'fast bootstrap' replicates. The BI analyses were performed using MRBAYES (v3.1.2; Ronquist & Huelsenbeck, 2003). Two independent Markov chain Monte Carlo (MCMC) runs were carried out with four differentially heated chains for 50 million generations, starting from a random tree. Model parameters were estimated during the analysis. Chains were sampled every 5000 generations. Convergence of the analyses was validated by (1) the standard deviation of the partition frequency [SD(s)] across runs (< 0.01); and (ii) monitoring the likelihood values over time using TRACER (v1.6.1; Drummond & Rambaut, 2007). The first 25% of generations were discarded as burn-in. A 50% majority-rule consensus tree was constructed from the remaining trees to estimate posterior probabilities (PPs).

MDA analysis

For each model analysis, goodness-of-fit based on the sum of squared deviations (*SSD*) and Harpending's (1994) raggedness index (H_{Rag}) was tested using parametric bootstrapping (Schneider & Excoffier, 1999) with 1000 pseudoreplicates. Their *P* values represented the probability of obtaining a simulated *SSD* value (or H_{Rag}) greater than or equal to the observed one. For two expanding lineages identified, the

MDA-derived expansion parameter (τ), and its 95% confidence interval (CI), were converted to absolute estimates of time since expansion using $\tau = 2ut$ (Rogers & Harpending, 1992; Rogers, 1995), where u is the neutral mutation rate for the entire sequence per generation (Won & Hey, 2005). The value for u was calculated as $u = \mu kg$, where μ is the substitution rate in substitutions per site per year (here, 1.3×10^{-9} s/s/y), k is the average sequence length of the cpDNA region in this study (here, 2669 bp; see the Results section), and g is the generation time in years [i.e. age of first reproduction; approximately three years, observed for *D. versipellis* in cultivation at the Botanic Garden of Zhejiang University (Qiu *et al.*, 2005)].

Detection of null alleles, HWE and LD

For the EST-SSR dataset, all 15 genotyped loci were checked for frequencies of null alleles in FREENA (Chapuis & Estoup, 2007) following the Expectation Maximization (EM) method described by Dempster *et al.* (1977). Deviations from Hardy-Weinberg equilibrium (HWE) were tested using GENEPOP (v4.0.7; Rousset, 2008). Linkage disequilibrium (LD) among loci was tested using FSTAT (v2.9.3; Goudet, 2001). Statistical significance (a = 0.05) for inferring LD or departures from HWE was evaluated based on 1000 permutations, and corrected for multiple tests using the sequential Bonferroni method (Rice, 1989).

STRUCTURE analysis

This program was run 10 times for a number of clusters (K), ranging from 2 to 40, using a burn-in length of 100,000 and run length of 500,000 iterations. We plotted the

probability of the data [lnP(*D*)] and the ad hoc statistic Δ*K* (Evanno *et al*., 2005) to determine the most likely *K*.

MIGRATE-N analysis

These analyses were run for three replicates assuming a stepwise mutation model (Estoup *et al*., 2002) with constant mutation rates for all loci. The initial run used an estimate of F_{ST} as a starting parameter to calculate *M* and each subsequent run used the ML estimates from the previous run as new starting parameters. For each run, we used uniform priors and Metropolis-coupled MCMC with 10 short chains and five long chains with 10,000 and 100,000 sampled genealogies, respectively, and set a static heating scheme at four temperatures (1, 1.3, 3 and 10). Genealogies were recorded every 50 steps and the first 10,000 were discarded as burn-in.

ABC analysis

For each of the eight models, we generated a reference table with 1×10^6 simulated data sets based on a divergence history that describes the model. All parameters that define each model (i.e. effective population size, divergence times and mutation rate) were considered as random variables with uniform distributions. For each simulation, the parameter values are drawn from their prior distributions, defining a divergence history that is used to construct a specific input file for the DIYABC program. The summary statistics of genetic variation were as follows: the mean number of alleles, mean expected heterozygosity (H_E) and mean allele size variance for each group, the overall F_{ST}, the classification indices (analogous to genotype assignment likelihoods),

and the shared allelic distance between pairs of groups. For both analyses, the prior distribution of mutation rates (μ, per generation per locus) for the EST-SSRs was assumed to be broad [1×10^{-6}, 1×10^{-3}] because they likely evolve more slowly than anonymous SSRs (Ellis & Burke, 2007). Likewise, a broad gamma distribution [1×10^{-1}, 9×10^{-1}] was specified for the parameter of the geometric distribution (P) to generate multiple stepwise mutations. However, for the effective population size and divergence time parameters, we employed different priors in the two steps (see details in Table 4). The average generation time was assumed to be three years (see MDA above). To compare the posterior probability of the four scenarios in each step, the 40,000 (1% proportion) simulated datasets closest to the observed dataset were selected for a polychotomous logistic regression approach (Cornuet *et al.*, 2008, 2010) and 4,000 for the direct estimate approach. For the best supported scenario, we estimated the posterior distributions of all parameters using a local linear regression on the 100,000 simulated datasets closest to the observed dataset, after the application of a logit transformation to parameter values (Beaumont *et al.*, 2002; Cornuet *et al.*, 2008). Finally, to evaluate whether the most fitting model successfully reproduced the observed data, we performed model-checking computations by simulating 100,000 pseudo-observed data sets from the posterior distribution of all parameters. Following Cornuet *et al.* (2010), we also conducted a principal component analysis (PCA) to test statistical vectors and visualize the consistency between simulated and observed data sets, using summary statistics that had not been used for model selection in the previous ABC treatments.

Supplementary Figures

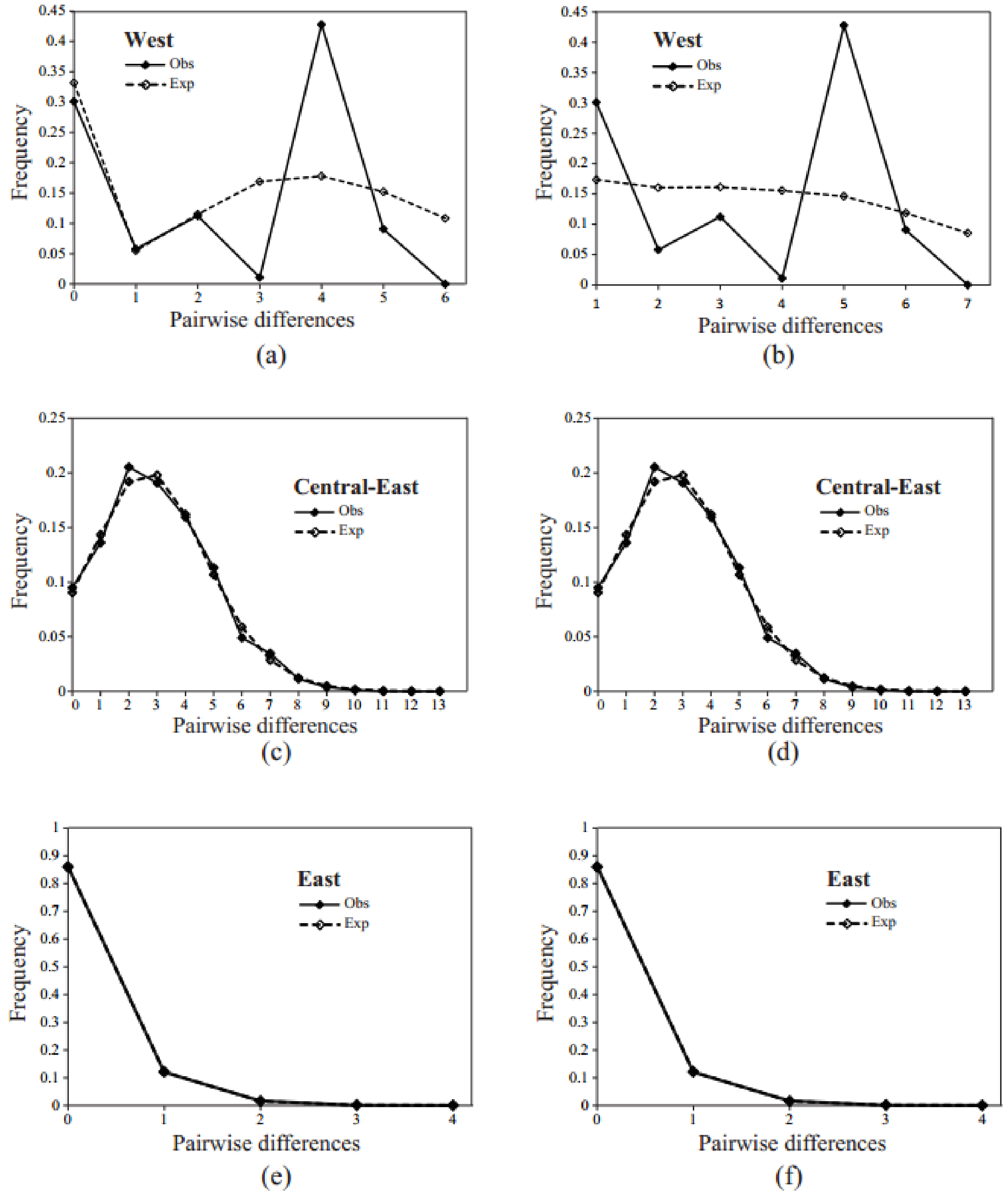

Figure S1. Mismatch distribution analyses (MDAs) of the three cpDNA lineages of the *Dysosma versipellis-pleiantha* complex (west, central-east: *D. versipellis s. lat.*; east: *D. pleiantha*) for the pure demographic expansion model (a, c, and e) and the spatial expansion model (b, d, and f).

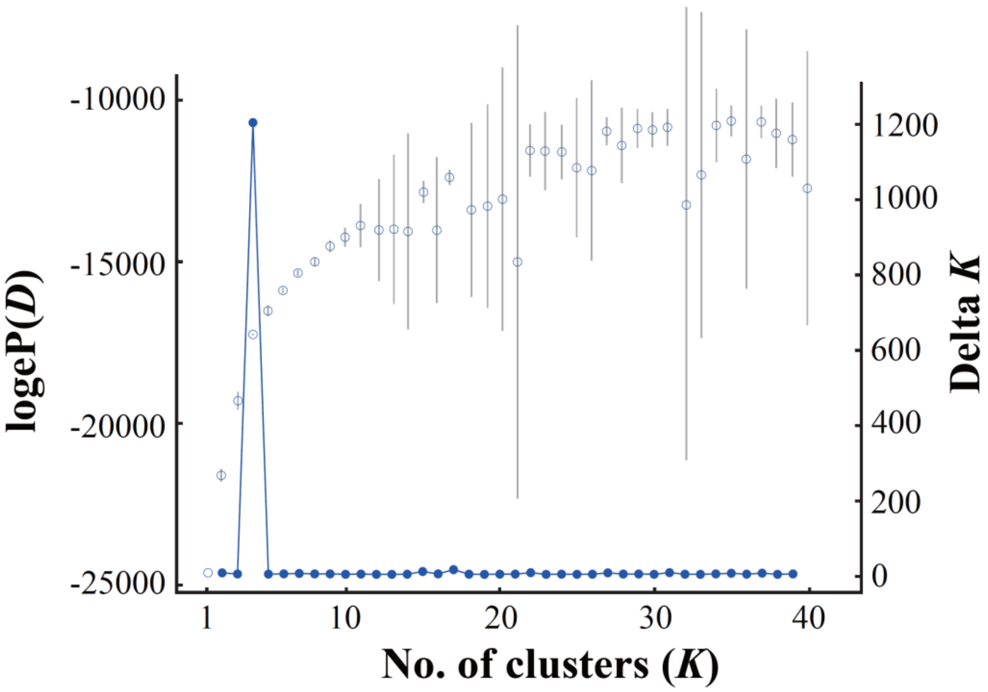

Figure S2. Changes of the mean posterior probability [ln P(*D*)] (±SD) values of each *K* calculated according to Pritchard *et al.* (2000) (dot plot); and the corresponding Δ*K* statistics calculated according to Evanno *et al.* (2005) (line diagram) in the STRUCTURE analysis based on 15 EST-SSR loci.

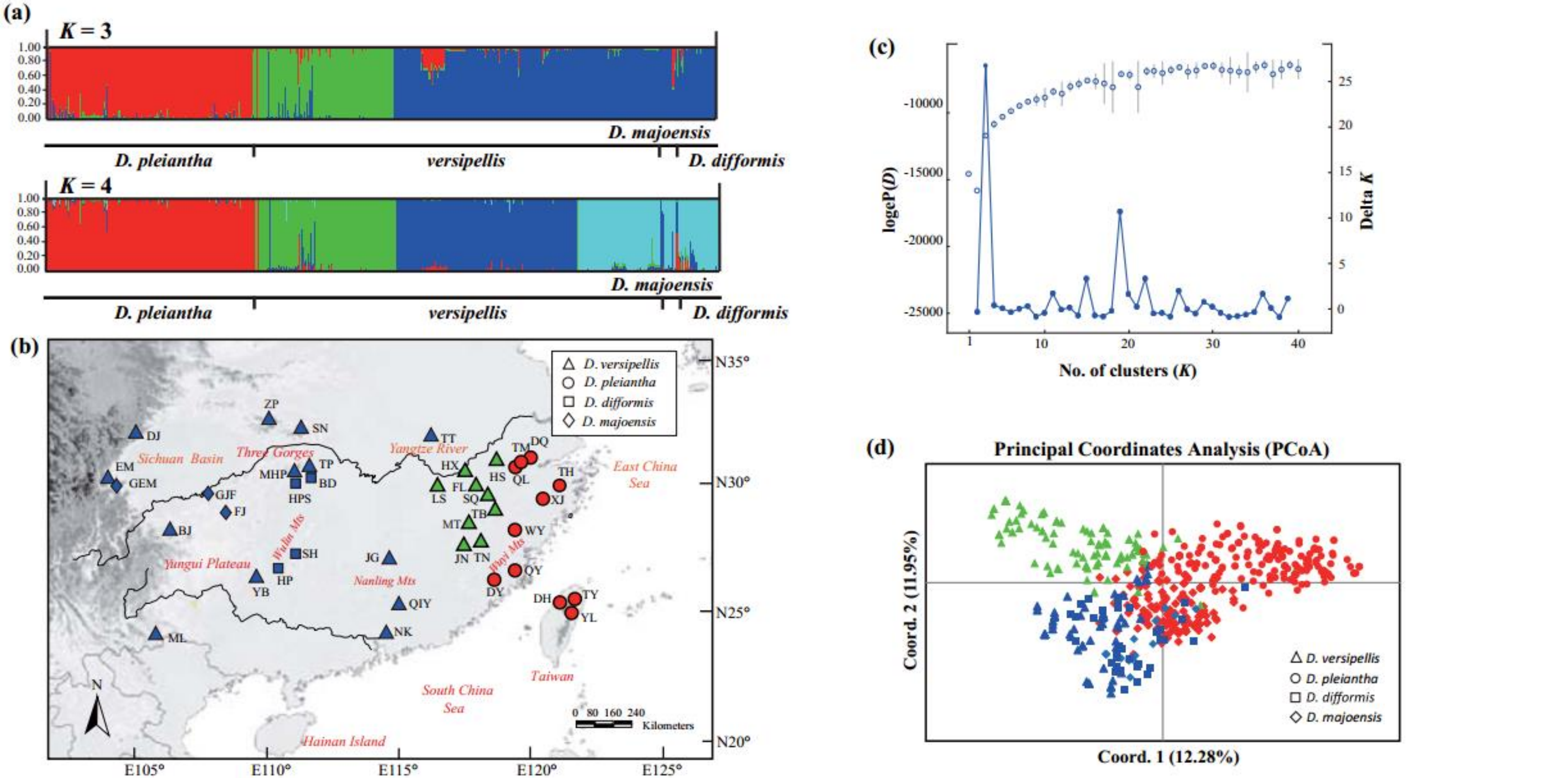
(a)
K = 3
1.00
0.80
0.60
0.40
0.20
0.00
D. majoensis
D. pleiantha
versipellis
D. difformis
K = 4
D. majoensis
D. pleiantha
versipellis
D. difformis
(b)
D. versipellis
D. pleiantha
D. difformis
D. majoensis
ZP
SN
DJ
TT
DQ
TM
QL
HS
HX
TH
EM
GEM
Sichuan Basin
Three Gorges
Yangtze River
East China Sea
MHP
TP
BD
HPS
GJF
FJ
FL
LS
SQ
XJ
TB
MT
WY
BJ
Wulin Mts
SH
JG
JN
TN
Wuyi Mts
Yungui Plateau
HP
YB
Nanling Mts
QY
DY
DH
TY
YL
QIY
ML
NK
South China Sea
Taiwan
Hainan Island
N
0 80 160 240
Kilometers
N35°
N30°
N25°
N20°
E105°
E110°
E115°
E120°
E125°
(c)
logeP(D)
Delta K
-10000
-15000
-20000
-25000
25
20
15
10
5
0
1
10
20
30
40
No. of clusters (K)
(d)
Principal Coordinates Analysis (PCoA)
Coord. 2 (11.95%)
Coord. 1 (12.28%)
D. versipellis
D. pleiantha
D. difformis
D. majoensis

Figure S3. (a) Results of the STRUCTURE assignment test at *K* = 3 and *K* = 4 for 40 populations of the *Dysosma versipellis-pleiantha* complex based on genetic variation at nine neutral EST-SSR loci. (b) Geographic origin of each *Dysosma* population and their color-coded grouping according to the STRUCTURE analysis at *K* = 3 created in ILLUSTRATOR v15.0 (http://www.adobe.com/products/illustrator.html). The symbol next to each sampling locality identifies the respective *Dysosma* species, while the filled color represents the cluster assigned to that population (population codes are identified in Table S2). The base map was drawn using ARCGIS v.9.3 (ESRI, Redlands, CA, USA). (c) Changes of the mean posterior probability [ln P(*D*)] (±SD) values of each *K* calculated according to Pritchard *et al.* (2000) (dot plot); and the corresponding Δ*K* statistics calculated according to Evanno *et al.* (2005) (line diagram) based on the nine neutral EST-SSR loci. (d) Principal coordinates analysis (PCoA) of the 577 individuals from the four species of the species complex based on EST-SSR variations (nine neutral loci).

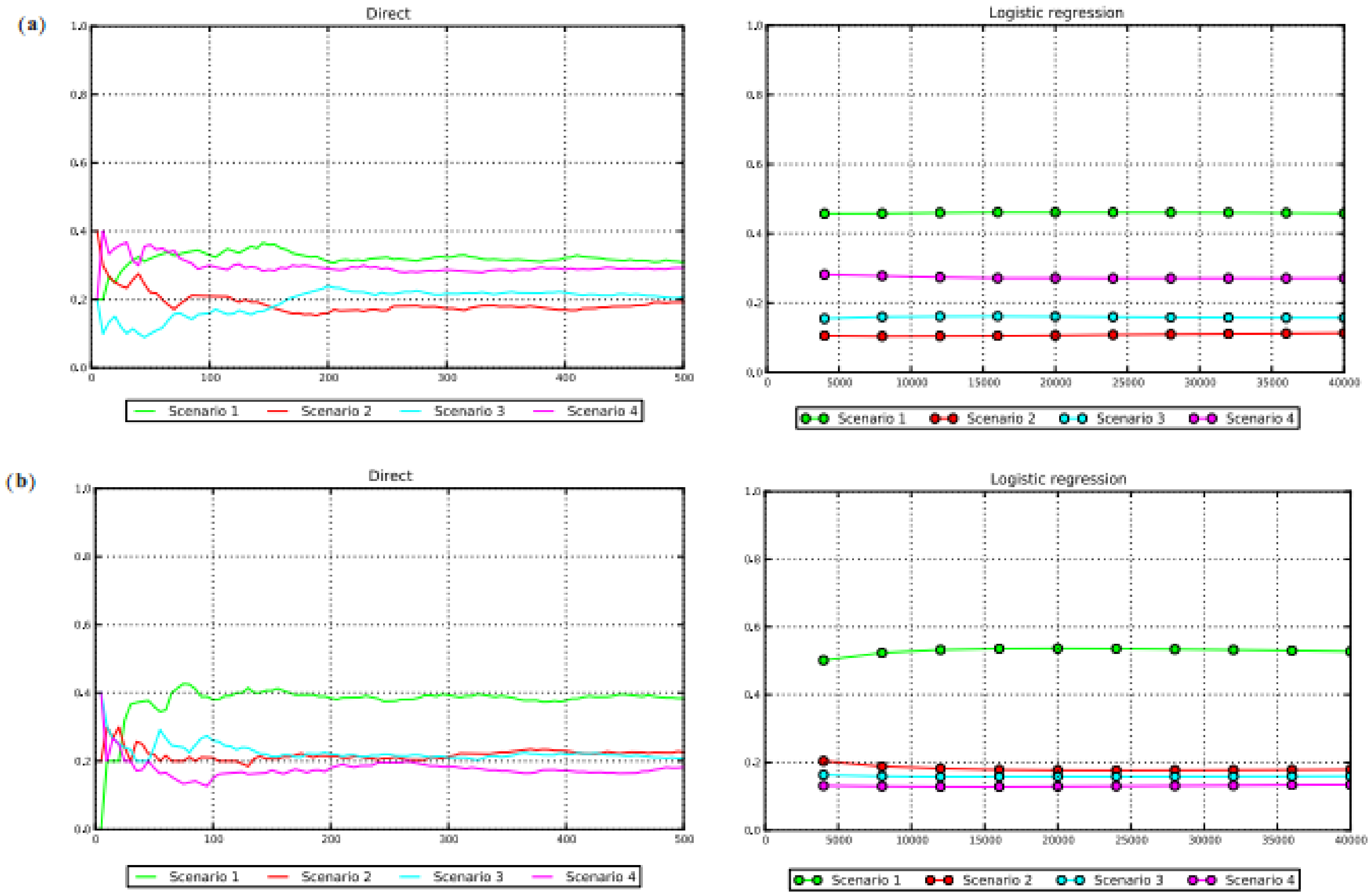

Figure S4. ABC model selection results using direct estimate and multinomial logistic regression approaches.

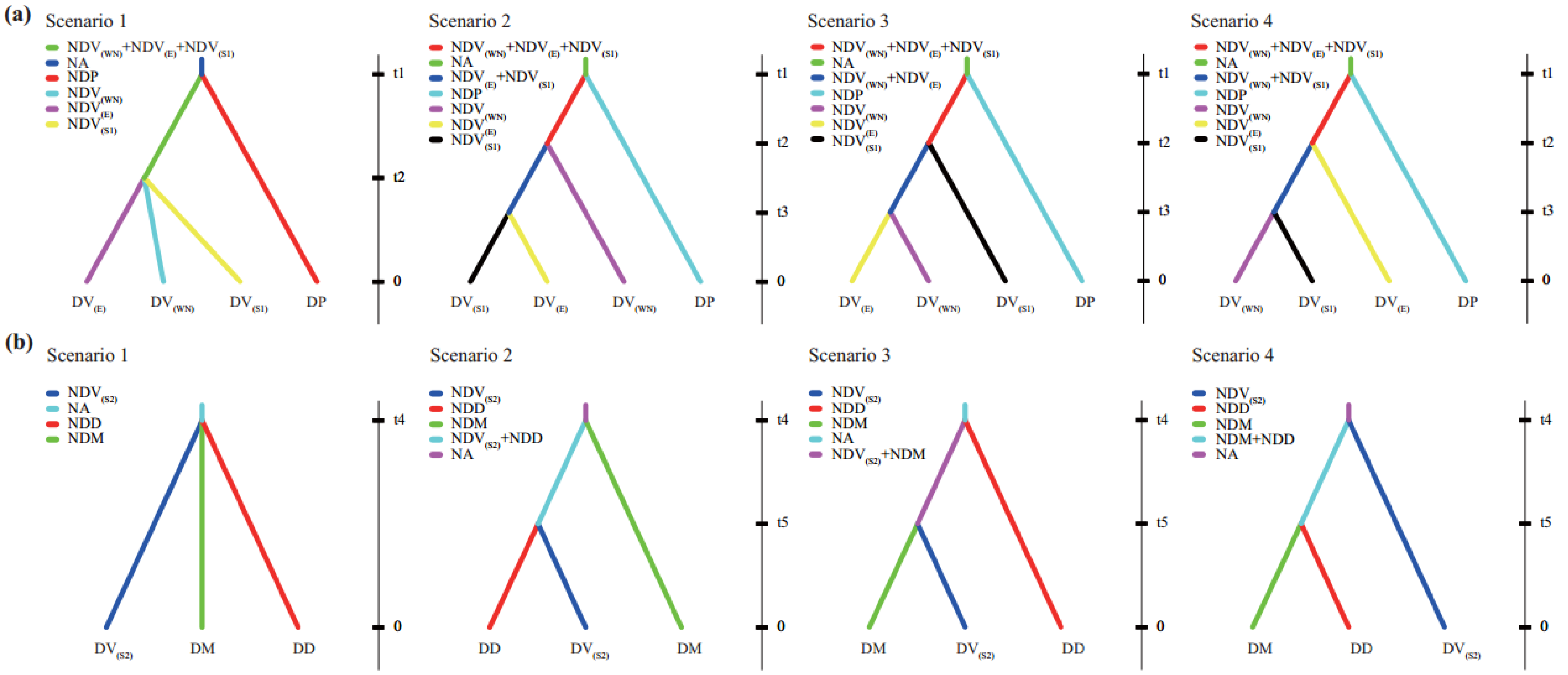

Figure S5. ABC modeling of scenarios for the diversification history of (a) the three regional EST-SSR (STRUCTURE) clusters of *Dysosma versipellis s. lat.*(west-north, east, south); and (b) the three taxonomic units of its 'southern cluster', i.e. *D. versipellis* (south), *D. difformis*

and *D. majoensis*. Divergence times (t_1–t_5; not to scale) are indicated and effective population sizes are marked in different colours. For parameter codes and scenario descriptions, see Table 4 and Supplementary Table S5, respectively.

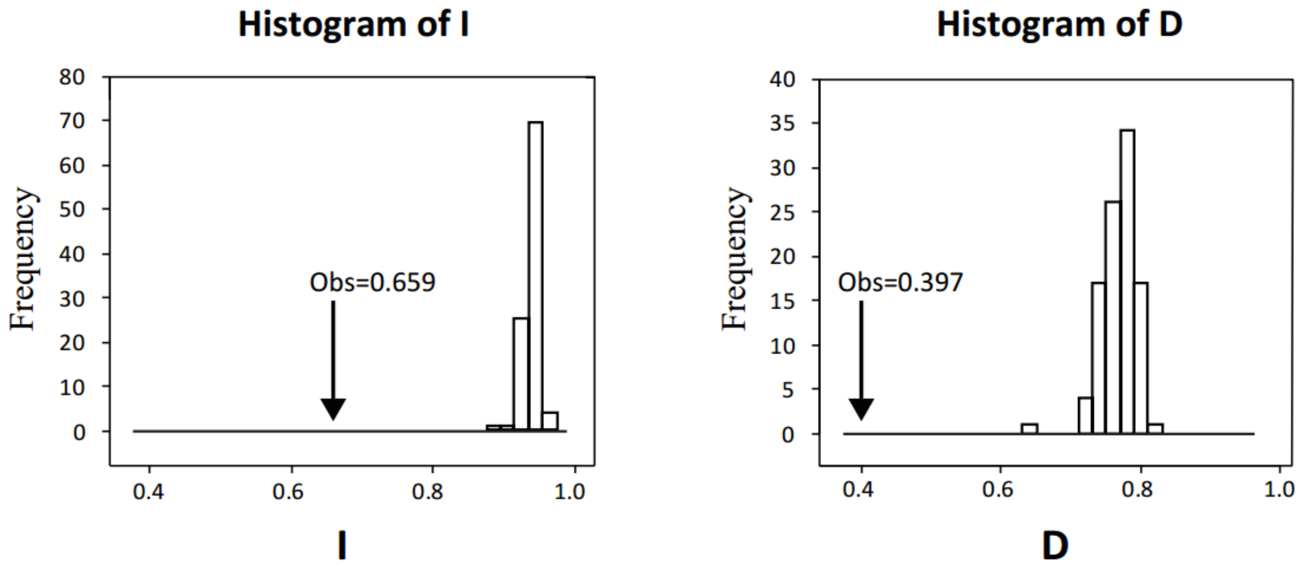

Figure S6. The results of identity tests between *Dysosma versipellis s. lat*. and *D. pleiantha* inhabiting, respectively, warm temperate deciduous (WTD) and warm temperate evergreen (WTE) forest in subtropical China. Bars indicate the null distributions of the standardized Hellinger distance (*I*) and Schoener's *D*, both generated from 100 randomizations. The x-axis indicates values of *I* and *D*, and the y-axis indicates the number of randomizations. The arrows indicate values of the actual MAXENT runs.

Supplementary Tables

Table S1. Chloroplast DNA sequence polymorphisms detected in three intergenic spacer regions of the *D. versipellis-pleiantha* complex and identifying 56 haplotypes (H1–56). All sequences are compared to the reference haplotype H1. Numbers 1/0 in sequences denote presence/absence of length polymorphism, identified by superscript letters (a–r). A dash (–) denotes a single nucleotide indel. a, ATTGGGGT; b, ATTAGGGT; c, AAGTTAGGA; d, TAACTAGTCTTT; e, TATTGGGGA; f, CTTCCAC; g, AATTGTAT; h, TATAGGTTATAGGT; i, TAATTA; j, TTGGGTGTCTAGAGATATGTCCTC; k, TTTGGTGTCTAGAGATATGTCCTC; l, CAAATA; m, AATGA; n, TGACTCCG; o, GTTATTAT; p, TAGATAT; q, GTAATAGAATCTATGATGAAGTAAT; r, GTAATAG.

	Nucleotide position																																																								
	*trn*L–*trn*F															*trn*F–*ndh*J																						*trn*S–*trnf*M																			
				1	1	2	2	3	3	3	5	6	7	8	8				2	2	2	3	3	4	4	4	4	5	5	6	6	6	6	6	7	7	7								4	5	6	6	6	6	6	6	7	7	7	8	9
		7	8	7	9	3	7	4	6	8	8	3	1	5	6	8	8	9	1	4	6	6	6	2	2	4	7	2	5	3	4	5	6	7	2	8	8	4	5	5	6	7	7	9	7	7	0	1	2	4	5	8	3	7	7	7	2
Haplotype	2	4	1	2	2	6	7	0	5	6	7	8	7	2	0	6	9	5	0	7	8	0	1	4	5	6	6	4	6	5	2	8	8	6	2	5	6	3	4	5	2	4	9	5	2	9	0	6	9	6	6	8	6	5	6	2	8
H1	T	G	T	C	C	G	1[a]	1[c]	0	0	0	0	0	G	A	T	0	A	1[j]	A	A	A	G	C	A	0	C	C	T	0	T	G	C	A	C	G	T	T	A	T	T	G	T	1[n]	T	C	T	0	A	A	1[p]	T	A	A	A	0	T
H2	·	·	·	·	·	·	1[b]	·	·	·	·	·	·	·	·	-	·	·	·	·	C	T	T	·	·	·	·	·	·	·	·	T	·	·	·	·	·	·	·	·	·	·	·	·	·	·	-	1[o]	·	·	·	·	·	·	·	·	·
H3	·	·	·	·	·	·	1[b]	·	·	·	·	·	·	·	·	-	·	·	·	·	C	T	T	·	·	·	·	·	·	·	·	T	·	·	·	·	·	·	·	·	·	·	·	·	·	·	-	·	·	·	·	·	·	·	·	·	·
H4	·	·	·	·	·	·	1[b]	0	·	·	·	·	·	·	·	-	·	·	·	·	·	T	T	·	·	·	·	·	·	·	·	T	·	·	·	·	·	·	·	·	·	·	·	·	·	·	-	·	·	·	·	·	·	·	·	·	·
H5	·	·	·	·	·	·	·	·	·	·	·	·	·	·	·	-	·	·	·	·	C	T	T	·	·	·	·	·	·	·	·	T	·	·	·	·	·	·	·	·	·	·	·	·	·	·	-	·	·	·	·	·	·	·	·	·	·
H6	·	·	·	T	·	·	1[b]	·	·	·	·	·	·	·	·	-	·	·	·	·	·	T	T	·	·	·	·	·	·	·	·	T	·	C	·	·	·	·	·	·	·	·	·	·	·	A	-	·	·	·	·	·	·	·	·	·	·
H7	·	·	·	·	·	·	·	·	·	·	·	·	·	·	·	-	·	·	·	·	·	T	T	·	·	·	·	·	·	·	·	T	·	C	·	·	·	·	·	·	·	·	·	·	·	A	-	·	·	·	·	·	·	·	·	·	·
H8	·	·	·	·	·	T	·	·	·	·	·	·	·	·	·	·	·	-	·	·	·	·	·	·	·	·	·	·	·	·	·	·	·	·	·	·	·	·	·	·	·	·	·	·	·	·	·	·	·	·	·	·	·	·	·	·	·

H9	·	·	·	·	·	·	·	·	·	·	·	·	·	·	·	·	·	-	·	·	·	·	·	·	·	·	·	·	·	·	·	·	·	·	·	·	·	·	·	·	·	·	·	·	·	·	·	·	·	·	·	·	·	·	·	·	·
H10	·	·	·	·	·	T	·	·	·	·	·	·	·	·	·	·	·	-	·	·	·	·	·	·	·	·	·	·	·	·	G	·	·	·	·	·	·	·	·	·	·	·	·	·	·	·	·	·	·	·	·	·	·	·	·	·	·
H11	·	·	·	·	·	·	·	·	·	·	·	·	·	·	·	·	·	·	·	·	·	·	·	·	·	·	·	·	·	·	·	·	T	·	·	·	·	·	·	·	·	·	·	·	·	·	·	·	·	·	·	·	·	·	·	·	·
H12	·	·	·	·	·	·	·	·	·	1e	·	·	·	·	·	·	·	·	-	·	·	·	·	·	·	·	·	·	·	·	·	·	·	·	·	·	·	·	·	·	·	·	·	·	·	·	·	·	·	·	·	·	·	·	·	·	·
H13	·	·	·	·	·	·	·	·	·	·	·	·	·	·	·	·	·	-	·	·	·	·	·	·	·	·	·	·	·	·	·	T	T	·	·	·	·	·	·	·	·	·	·	·	·	·	·	·	·	·	·	·	·	·	·	·	·
H14	·	·	·	·	·	·	·	·	·	·	·	·	·	·	·	·	·	-	·	·	·	·	·	·	·	·	·	·	·	·	·	·	T	·	·	·	·	·	·	·	·	·	·	·	·	·	·	·	·	·	·	·	·	·	·	·	·
H15	·	·	·	·	·	·	·	·	·	·	·	·	·	·	·	·	·	-	·	·	·	·	·	·	·	·	·	·	·	·	G	·	T	·	·	·	·	·	·	·	·	·	·	·	·	·	·	·	·	·	·	·	·	·	·	·	·
H16	·	·	·	T	·	·	1b	·	·	·	·	·	·	·	·	·	·	-	·	·	·	·	·	·	·	·	·	·	·	·	·	·	·	·	·	·	·	·	·	·	·	·	·	·	·	·	·	·	·	·	·	·	·	·	·	·	·
H17	·	·	·	·	·	·	·	·	·	·	·	·	·	·	·	·	·	·	·	·	·	·	·	·	·	·	·	·	·	·	·	·	·	·	·	·	·	·	·	·	·	·	·	·	·	·	·	·	·	·	·	·	·	·	·	1^{q}	·
H18	G	·	·	·	T	·	·	·	·	·	1f	·	·	·	·	·	·	-	·	·	·	·	·	T	·	·	·	·	·	·	·	·	·	·	·	·	·	·	·	·	·	·	·	·	·	·	·	·	·	·	·	·	·	·	·	·	·
H19	·	·	·	·	·	·	0	·	·	·	·	·	·	·	·	·	·	-	1^{k}	·	·	·	·	·	·	·	·	·	·	·	·	·	·	·	·	·	·	·	·	·	·	·	·	·	·	·	·	·	·	·	·	·	·	·	·	·	·
H20	·	·	·	·	·	·	0	·	·	·	·	·	·	·	·	·	·	-	1^{k}	·	·	·	·	·	·	·	·	·	·	·	·	·	·	·	·	·	·	·	G	·	G	·	G	·	·	·	·	·	·	·	·	·	·	·	·	·	·
H21	·	·	·	·	·	·	0	·	·	·	·	·	·	·	·	·	·	·	1^{k}	·	·	·	·	·	·	·	·	·	·	·	·	·	·	·	·	·	·	·	·	·	·	·	·	·	·	·	·	·	·	·	·	·	·	·	·	·	·
H22	·	·	·	·	·	·	0	·	·	·	·	·	·	·	·	·	·	·	1^{k}	·	·	·	A	·	·	1^{l}	·	·	·	·	·	·	·	·	·	·	·	·	·	·	·	·	·	·	·	·	·	·	·	·	·	·	·	·	·	·	·
H23	·	·	·	·	·	·	·	·	·	·	·	·	·	·	·	·	·	·	·	·	·	·	·	·	·	·	·	·	·	·	·	·	·	·	·	-	-	·	·	·	·	·	·	·	·	·	·	·	·	·	·	·	·	·	·	·	·
H24	·	·	·	·	·	·	·	·	·	·	·	·	·	-	·	·	·	·	·	·	·	·	·	·	·	·	·	·	·	·	·	·	·	·	·	·	·	·	·	·	·	·	·	·	·	·	·	·	·	·	·	·	·	·	·	·	·
H25	·	·	G	·	·	·	·	·	·	·	·	·	·	·	·	·	·	·	·	·	·	·	·	·	·	·	·	·	·	·	·	·	·	·	·	·	·	·	·	·	·	·	·	·	·	·	·	·	·	·	·	·	·	·	·	·	·
H26	·	·	G	·	·	·	·	·	·	·	·	·	·	·	·	·	·	-	·	·	·	·	·	·	·	·	·	·	·	·	·	·	·	·	·	·	·	·	·	·	·	·	·	·	·	·	·	·	·	·	·	·	·	·	·	·	·
H27	·	·	·	·	·	·	·	·	·	·	·	·	·	·	·	·	·	-	·	·	·	·	·	·	·	·	·	T	·	·	·	·	·	·	·	·	·	·	·	·	·	·	·	·	·	·	·	·	·	·	·	·	·	·	·	·	·
H28	·	·	·	·	·	·	·	·	·	·	·	·	·	·	·	·	·	-	·	·	·	·	·	·	·	1^{l}	·	·	·	·	·	·	·	·	·	·	·	·	·	·	·	·	·	·	·	·	·	·	C	·	·	·	·	·	·	·	·
H29	·	·	·	·	·	·	·	·	·	·	·	·	·	·	·	·	·	-	·	·	·	·	A	·	C	1^{l}	·	·	·	·	·	·	·	·	·	·	·	·	·	·	·	·	·	·	·	·	·	·	C	·	·	·	·	·	·	·	·
H30	·	·	·	·	·	·	·	·	·	·	·	·	·	·	·	·	·	-	·	·	·	·	A	·	·	1^{l}	·	·	·	·	·	·	·	·	·	·	·	·	·	·	·	·	·	·	·	·	·	·	C	·	·	·	·	·	·	·	·
H31	·	·	·	·	·	·	·	·	·	·	·	·	·	·	·	·	·	·	·	·	·	·	A	·	·	1^{l}	·	·	·	·	·	·	·	·	·	·	·	·	·	·	·	·	·	·	·	·	-	·	·	C	·	·	·	·	·	·	·

H32	·	·	·	·	·	·	·	·	·	·	·	·	·	·	·	·	·	·	·	·	·	·	A	·	C	1l	·	·	·	·	·	·	·	·	·	·	·	·	·	·	·	·	·	·	·	·	·	·	C	·	·	·	·	·	·	·	·
H33	·	·	·	·	·	·	·	·	·	·	·	·	·	·	·	·	·	·	·	·	·	·	A	·	·	1l	·	·	·	·	·	·	·	·	·	·	·	·	·	·	·	·	·	·	·	·	·	·	C	·	·	·	·	·	·	·	·
H34	·	·	·	·	T	·	·	·	·	·	1f	·	·	·	·	·	·	-	·	·	·	·	A	·	C	1l	·	·	·	·	·	·	·	·	·	·	·	·	·	·	·	·	·	·	·	·	·	·	C	·	·	·	·	·	·	·	·
H35	·	·	·	T	·	·	1b	·	·	·	·	·	·	·	·	-	·	·	·	·	·	T	T	·	·	·	·	·	·	·	·	T	·	C	·	·	·	·	·	·	·	·	·	·	·	·	·	·	·	·	·	·	·	·	·	·	·
H36	·	·	·	·	·	·	·	·	·	·	·	·	·	·	·	·	1i	·	·	C	·	·	·	·	·	·	·	·	·	·	·	·	·	·	·	·	·	·	·	·	·	·	·	·	·	·	·	·	·	·	·	·	·	·	·	·	·
H37	·	·	·	·	·	·	·	·	·	·	·	·	·	·	·	·	·	·	·	·	·	·	·	·	·	·	·	·	·	·	·	·	·	·	·	·	·	·	·	·	·	·	·	·	G	·	·	·	·	·	·	·	·	·	·	·	·
H38	·	·	·	·	·	·	·	·	·	·	·	·	·	·	·	·	·	·	·	·	·	·	·	·	·	·	·	·	·	·	·	·	·	·	·	·	·	·	·	·	·	·	·	·	·	·	·	·	·	·	·	·	·	C	G	·	·
H39	·	·	·	·	·	·	·	·	·	·	·	·	·	·	·	·	·	·	·	·	·	·	·	·	·	·	·	·	·	·	·	·	·	·	·	·	·	·	·	·	·	·	·	0	·	·	·	·	·	·	·	·	·	·	·	·	·
H40	·	·	·	·	·	·	·	·	·	·	·	·	·	·	-	·	·	·	·	·	·	·	·	·	·	·	·	·	·	·	·	·	·	·	·	·	·	·	·	·	·	·	·	0	·	·	·	·	·	·	·	·	·	·	·	·	·
H41	·	·	·	·	·	·	·	·	·	·	·	·	·	·	·	·	·	·	·	·	·	·	·	·	·	·	·	·	·	·	·	·	·	·	·	·	·	-	·	·	·	·	·	0	·	·	·	·	·	·	·	·	·	·	·	·	·
H42	·	·	·	·	·	·	·	·	·	·	·	·	·	·	·	·	·	·	·	·	·	·	·	·	·	·	·	·	·	·	·	·	·	·	A	·	·	·	·	·	·	·	·	·	·	·	·	·	·	·	0	·	·	·	·	·	·
H43	·	·	·	·	·	·	·	·	·	·	·	1g	·	·	·	·	·	·	·	·	·	·	·	·	·	·	·	·	·	·	·	·	·	·	·	·	·	·	·	·	·	A	·	·	·	·	·	·	·	·	·	·	·	·	·	·	·
H44	·	·	·	·	·	·	·	·	·	·	·	1g	·	·	·	·	·	·	·	·	·	·	·	·	·	·	·	·	·	·	·	·	·	·	·	·	·	·	·	·	·	·	·	·	·	·	·	·	·	·	·	·	·	·	·	·	·
H45	·	·	·	·	·	·	·	·	·	·	·	·	·	·	·	·	·	·	·	·	·	·	·	·	·	·	·	·	·	·	·	·	·	·	·	·	·	·	·	·	·	A	·	·	·	·	·	·	·	·	·	·	·	·	·	·	·
H46	·	·	·	·	·	·	·	·	·	·	·	·	·	·	·	·	·	·	·	·	·	·	·	·	·	·	·	·	·	·	·	·	·	·	·	·	·	·	·	-	·	·	·	·	·	·	·	·	·	·	·	·	·	·	·	·	·
H47	·	A	·	·	·	·	1b	·	·	·	·	·	·	·	·	-	·	·	·	·	·	·	T	·	·	·	·	·	·	·	·	T	·	·	·	·	·	·	·	·	·	·	·	·	·	·	-	·	·	C	·	·	C	·	·	·	·
H48	·	A	·	·	·	·	1b	·	·	·	·	·	·	·	·	-	·	·	·	·	·	·	T	·	·	·	·	·	·	·	·	T	·	·	·	·	·	·	·	·	·	·	·	·	·	·	-	·	·	C	·	·	·	·	·	·	·
H49	·	A	·	·	·	·	1b	·	·	·	·	·	·	·	·	-	·	·	·	·	·	·	T	·	·	·	·	·	·	·	·	T	·	·	A	·	·	·	·	·	·	·	·	·	·	·	-	·	·	C	·	·	·	·	·	·	·
H50	·	A	·	·	·	·	1	·	1	·	·	·	·	·	·	-	·	·	·	·	·	·	T	·	·	·	·	·	·	·	·	T	·	·	A	·	·	·	·	·	·	·	·	·	·	·	-	·	·	C	·	·	·	·	·	·	·

							b		d																																																
H51	·	A	·	·	·	·	1[b]	·	·	·	·	·	·	·	·	-	·	·	·	·	·	·	T	·	·	·	·	·	-	·	·	T	·	·	·	·	·	·	·	·	·	·	·	·	·	·	-	·	·	C	·	·	·	·	·	·	·
H52	·	·	·	·	·	·	·	·	·	·	·	·	·	·	·	·	·	·	·	·	·	·	·	·	·	·	·	·	·	·	·	·	·	·	·	·	·	·	·	·	·	·	·	·	·	·	·	·	·	·	·	·	·	·	·	1[r]	·
H53	·	·	·	·	·	·	·	·	·	·	·	·	·	·	·	·	·	·	·	·	·	·	·	·	·	·	·	·	·	·	·	·	·	·	·	·	·	·	·	·	·	·	·	·	·	·	·	·	·	·	·	C	·	·	·	1[r]	·
H54	·	·	·	·	·	·	·	·	·	·	·	·	·	·	·	·	·	·	·	·	·	·	·	·	·	·	·	·	·	1[m]	·	·	·	·	·	·	·	·	·	·	·	·	·	·	·	·	·	·	·	·	·	C	·	·	·	1[r]	·
H55	·	·	·	·	·	·	·	·	·	·	·	·	·	·	·	·	·	·	·	·	·	·	·	·	·	·	A	·	·	·	·	·	·	·	·	·	·	·	·	·	·	·	·	·	·	·	·	·	·	·	·	·	·	·	·	·	·
H56	·	·	·	·	·	·	·	·	·	·	·	·	1[h]	·	·	·	·	·	·	·	·	·	·	·	·	·	·	·	·	·	·	·	·	·	·	·	·	·	·	·	·	·	·	0	·	·	·	·	·	·	·	·	·	·	·	·	C

Table S2. Geographic and genetic characteristics of *Dysosma* population samples used in this study. *n*, number of individuals; *h*, haplotype diversity; *π*, nucleotide diversity; N_A, number of alleles; A_R, allele richness; H_E, expected heterozygosity; F_{IS}, fixation index.

Species/ Population code	Locality	Latitude (N)	Longitude (E)	Altitude (m)	*n* (cpDNA/EST-SSR)	cpDNA *h* (±SD)	cpDNA $\pi \times 10^{-3}$ (±SD)	cpDNA haplotypes	EST-SSR (All 15 loci/nine neutral loci) N_A	A_R	H_E	F_{IS}
Dysosma versipellis												
EM	Mt. E’mei, Sichuan Province	29º35'	103º21'	1200	12/23	0.167	0.13	H6, H7	44/28	1.274/1.307	0.274/0.307	-0.014/-0.102
DJ	Dujiangyan, Sichuan Province	31º06'	103º38'	1290	11/21	0.491	0.29	H2–H5	38/25	1.403/1.393	0.396/0.386	-0.756/-0.714
JF	Mt. Jinfo, Chongqing City	29º10'	107º10'	1650	12/–	0.167	0.60	H35, H36	–	–	–	–
ML	Malipo, Yunnan Province	23°07'	104°45'	1805	11/11	0.182	0.07	H1, H46	24/13	1.102/1.105	0.101/0.103	-0.138/-0.373
NG	Mt. Nangong, Shanxi Province	32º15'	109º04'	1800	10/–	0.556	0.34	H19, H20	–	–	–	–
SN	Shennongjia, Hubei Province	31º28'	110º22'	2000–2700	10/33	0	0	H18	56/39	1.457/1.496	0.459/0.497	0.212/0.161
TP	Mt. Tianping, Hunan Province	29º08'	110º28'	1000	10/14	0.712	0.50	H8, H9, H26, H27	38/24	1.403/1.422	0.402/0.421	-0.077/-0.055
TT	Tiantangzhai, Anhui Province	31º14'	115º74'	900–1200	18/36	0.837	0.74	H28–H34	47/29	1.257/1.292	0.257/0.292	-0.029/-0.061
HS	Mt. Huang, Anhui Province	30º08'	118º18'	800–1000	10/16	0.757	0.93	H1, H11–H15	37/24	1.275/1.316	0.275/0.316	0.028/0.012

HX	Haixing, Jiangxi Province	29º95'	116º87'	800	12/11	0.318	0.20	H9, H14, H16	36/24	1.293/1.386	0.296/0.391	0.219/0.246
FL	Fuliang, Jiangxi Province	29º55'	117º66'	800	10/2	0.511	0.22	H8–H10	25/16	1.344/1.463	0.304/0.406	0.059/0.077
SQ	Mt. Sanqing, Jiangxi Province	28º54'	118º03'	800	18/26	0.386	0.28	H1, H23, H24	44/31	1.350/1.468	0.351/0.470	0.174/0.190
TB	Mt. Tongba, Jiangxi Province	28º05'	118º15'	700–900	8/8	0	0	H1	22/14	1.203/1.299	0.192/0.278	-0.783/-1.000
JG	Mt, Jinggang, Jiangxi Province	26º28'	114º13'	300–500	11/11	0	0	H37	26/17	1.212/1.226	0.208/0.222	-0.422/-0.354
QIY	Mt, Qiyun, Jiangxi Province	25º46'	113º57'	570	14/13	0.495	0.43	H38–H41	31/24	1.276/1.403	0.274/0.401	-0.200/-0.130
NK	Mt. Nankun, Guangdong Province	32º15'	109º04'	1800	17/19	0	0	H42	28/18	1.195/1.165	0.195/0.165	-0.093/-0.015
YB	Mt. Yuanbao, Guangxi Province	25º26'	109º09'	1400	14/17	0	0	H1	38/22	1.374/1.382	0.373/0.384	-0.063/0.166
JN	Jianning, Fujian Province	26°55'	116°41'	500–700	19/29	0.368	0.15	H43–H45	37/24	1.215/1.208	0.216/0.209	0.048/0.223
MHP	Mt. Huping, Hunan Province	30°01'	110°32'	900–1200	7/11	0	0	H1	32/20	1.221/1.199	0.225/0.203	0.381/0.401
LS	Mt. Lu, Jiangxi Province	29°32'	115°59	1082	10/11	0.644	0.37	H1, H12, H17	38/25	1.295/1.319	0.299/0.322	0.249/0.216
TN	Taining, Fujian Province	26°54'	117°10'	700	15/16	0	0	H43	30/19	1.157/1.162	0.157/0.162	-0.091/0.013
MT	Mt. Matou,	27º48'	117º08'		-/3	–	–	–	28/19	1.291/1.374	0.300/0.389	0.074/0.095

	Jiangxi province											
ZP	Zhenping county, Shanxi province	31°59'	109°20'		-/17	–	–	–	19/12	1.080/1.076	0.078/0.075	-0.856/-0.749
BJ	Bijie, Guizhou Province	27º24'	105º54'	1697	3/2	0	0	H56	18/11	1.133/1.148	0.100/0.111	-1.000/-1.000
Species mean						**0.300 (0.29)**	**0.239 (0.27)**		33/21	1.264/1.300	0.261/0.296	-0.140/-0.125
Species total						**0.902**	**1.42**		127/80	6．403/6.795	0.619/0.635	0.529/0.503
Dysosma pleiantha												
TM	Mt. Tianmu, Zhejiang Province	30º19'	119º26'	400	19/26	0.450	0.22	H48–H51	58/42	1.412/1.464	0.414/0.465	0.188/0.141
DQ	Deqing, Zhejiang Province	28º54'	118º03'	400–500	9/36	0	0	H48	43/26	1.319/1.337	0.320/0.337	0.172/0.086
TH	Mt. Tiantai, Zhejiang Province	29º14'	120º00'	500	5/17	0	0	H47	42/31	1.372/1.489	0.374/0.492	0.152/0.158
XJ	Xianju, Zhejiang Province	28º42'	120º36'	500	8/8	0	0	H48	18/12	1.101/1.168	0.094/0.157	-0.765/-0.765
WY	Wuyanling, Zhejiang Province	27º41'	119º40'	1065	10/14	0	0	H48	39/26	1.323/1.387	0.323/0.385	0.030/-0.147

DY	Mt. Daiyun, Fujian Province	25º38'	118º11'	300	21/24	0	0	H48	45/33	1.320/1.481	0.320/0.481	0.045/0.027
QY	Mt. Qingyun, Fujian Province	26º01'	118º55'	600	19/23	0	0	H48	30/20	1.292/1.410	0.287/0.403	-0.770/-0.811
TY	County Taoyuan, Taiwan	24º43'	121º26'	1995	6/5	0	0	H48	26/15	1.289/1.267	0.287/0.267	-0.070/0.000
YL	County Yilan, Taiwan	24º34'	121º24'	1594/1699	18/13	0	0	H48	33/22	1.343/1.435	0.343/0.434	0.005/-0.064
DH	Mt. Dahun, Taiwan, China	24°41'	121°17'	1520	9/8	0	0	H48	37/26	1.362/1.468	0.358/0.460	-0.165/-0.237
QL	Mt. qingliang, Anhui province	30 º06'	118°54'	600	-/4	–	–	–	25/17	1.258/1.333	0.298/0.434	0.032/0.123
Species mean						**0.045 (0.14)**	**0.02 (0.07)**		36/25	1.308/1.385	0.311/0.392	-0.104/-0.135
Species total						**0.154**	**0.07**		85/60	3.347/4.116	0.473/0.541	0.296/0.180
Dysosma difformis												
HP	Huaping, Guangxi Province	25º38'	109º54'	700	8/8	0	0	H1	45/29	1.481/1.494	0.500/0.507	0.491/0.381
HPS	Mt. Huping, Hunan Province	30º01'	110º32'	925	4/3	0.500	0.20	H1, H55	22/14	1.200/1.229	0.200/0.241	0.000/0.231

BD	Mt. Tianping, Hunan Province	29º45'	110º03'	1513	8/8	0	0	H55	33/20	1.419/1.455	0.412/0.444	-0.254/-0.375
SH	Mt. Shunhuang, Hunan Province	26º26'	110º58'	993	15/16	0.657	0.36	H1, H52–H54	52/31	1.431/1.435	0.432/0.439	0.070/0.280
Species mean						**0.289 (0.34)**	**0.14 (0.17)**		38/24	1.383/1.403	0.386/0.408	0.077/0.129
Species total						**0.696**	**0.40**		74/45	4.879/4.974	0.610/0.627	0.377/0.410
Dysosma majoensis												
FJ	Mt. Fanjing, Guizhou Province	27º56'	108º36'	942	6/6	0	0	H55	30/21	1.322/1.379	0.336/0.393	0.404/0.387
GJF	Mt. Jinfo, Chongqing	29º00'	107º11'	1823	5/5	0	0	H55	28/16	1.202/1.146	0.203/0.147	0.082/0.094
GEM	Mt. E'mei, Sichuan Province	29°35'	103°22'	2200	3/3	0	0	H55	24/16	1.289/1.311	0.244/0.305	0.122/-0.091
Species mean						0	0		27/18	1.271/1.278	0.261/0.282	0.203/0.130
Species total						0	0		48/33	3.014/3.548	0.447/0.464	0.523/0.512
**All total**						**0.929**	**3.58**		**138/85**	**5.064/5.589**		

Table S3. BEAST-derived estimates of T_{MRCA} and their 95% heighest posterior densities (HPDs) under two coalescent models (constant population-size and population expansion models) for relevant nodes of the Bayesian inference (BI) phylogeny of *Dysosma* (plus outgroups) based on cpDNA sequence data (see Fig. 2).

		Model	
Node code	Descriptions of the corresponding clade	Constant size (HPD 95%)	Expansion growth (HPD 95%)
Node A	*Dysosma* clade	4.14 (2.74−5.44) Ma	3.44 (2.30−4.68) Ma
Node B	*D. delavayi* and *D. versipellis-pleiantha* complex clade	3.33 (2.15−4.26) Ma	2.66 (1.74−3.63) Ma
Node C	*D. versipellis-pleiantha* complex	2.16 (1.33−2.72) Ma	1.66 (1.42−3.07) Ma
Node D	Western and eastern lineages	1.61 (0.81−2.09) Ma	1.23 (0.74−1.73) Ma
Node E	Central-eastern lineage	1.36 (0.81−1.68) Ma	1.02 (0.70−1.36) Ma
Node F	Western lineage	0.91 (0.33−1.27) Ma	0.70 (0.39−1.07) Ma
Node G	Eastern lineage	0.59 (0.11−0.88) Ma	0.52 (0.25−0.83) Ma

Table S4. Selective outliers identified by different F_{ST}-based neutrality test methods as implemented in LOSITAN, BAYESCAN and ARLEQUIN. Selective outliers are denoted by a plus sign (+) when the *P*-value was < 0.05. Following a Bonferroni correction, selective outliers exhibiting significance are indicated by an asterisk (*).

Outlier detection method	Locus					
	Positive outlier			Balancing outlier		
	EDV-46	EDV-52	EDV-119	EDV-60	EDV-67	EDV-82
LOSITAN	+	*	+	*	*	*
BAYESCAN	*	*		+		
ARLEQUIN	*	*	+	*	*	+

Table S5. Four scenarios each tested by DIYABC modeling for the diversification history of (a) the three regional EST-SSR (STRUCTURE) clusters of *D. versipellis s. lat.* (west-north, east, south); and (b) the three taxonomic units of its 'southern cluster', i.e. *D. versipellis* (south), *D. difformis*, and *D. majoensis*. See also Supplementary Fig. S5 for scenario illustrations. Note that *D. pleiantha* was generally assumed as sister, based on the cpDNA haplotype network analysis (see Results, Fig. 1c). In (a), DP, $DV_{(WN)}$, $DV_{(E)}$, and $DV_{(S1)}$ represent, respectively, *D. pleiantha*, *D. versipellis* west-north cluster, *D. versipellis* east cluster and *D. versipellis* south cluster (including *D. difformis* and *D. majoensis*). In (b), $DV_{(S2)}$, DD, and DM represent, respectively, *D. versipellis* south cluster, *D. difformis* and *D. majoensis.*

Scenario	Description of divergence scenarios
(a)	
Scenario 1	DP and a common ancestor of $DV_{(E)}$, $DV_{(S1)}$ and $DV_{(WN)}$ diverged at t_1, and then $DV_{(E)}$, $DV_{(S1)}$ and $DV_{(WN)}$ diverged at t_2 simultaneously.
Scenario 2	DP and a common ancestor of $DV_{(E)}$, $DV_{(S1)}$ and $DV_{(WN)}$ diverged at t_1, and then $DV_{(WN)}$ and a common ancestor of $DV_{(S1)}$ and $DV_{(E)}$ diverged at t_2, finally, DM and DD diverged at t_3.
Scenario 3	DP and a common ancestor of $DV_{(E)}$, $DV_{(S1)}$ and $DV_{(WN)}$ diverged at t_1, and then $DV_{(S1)}$ and a common ancestor of $DV_{(E)}$ and $DV_{(WN)}$ diverged at t_2, finally, $DV_{(E)}$ and $DV_{(WN)}$ diverged at t_3.
Scenario 4	DP and a common ancestor of $DV_{(E)}$, $DV_{(S1)}$ and $DV_{(WN)}$ diverged at t_1, and then $DV_{(E)}$ and a common ancestor of $DV_{(S1)}$ and $DV_{(WN)}$ diverged at t_2, finally, $DV_{(S1)}$ and $DV_{(WN)}$ diverged at t_3.
(b)	
Scenario 1	$DV_{(S2)}$, DM and DD diverged at t_4 simultaneously.
Scenario 2	DM and a common ancestor of DD and $DV_{(S2)}$ diverged at t_4, then DD and $DV_{(S2)}$ diverged at t_5.
Scenario 3	DD and a common ancestor of DM and $DV_{(S2)}$ diverged at t_4, then DM and $DV_{(S2)}$ diverged at t_5.
Scenario 4	$DV_{(S2)}$ and a common ancestor of DD and DM diverged at t_4, then DD and DM diverged at t_5.

Table S6. Primer information of the five newly-developed EST-SSR markers used in this study.

Locus	Forward primer sequences (5′—3′)	Reverse primer sequences (5′—3′)	Repeated motif	Size range (bp)
EDV-81	CTCTGACGAATCATCCTCCTCTA	ACATTATGCTAGTGATGGCGTTT	$(TTC)_5$	154–172
EDV-82	TACTGAGGGAGTTCCATGAGATG	CAACTCAAACCTTCTTCTGGGTA	$(GGT)_5$	159–186
EDV-102	AGACGCTATAGAGTTTTACCGCC	ATCTTGAATTGAACCAGTCCGTA	$(TGG)_5$	146–167
EDV-118	AGAGAGCTGATGCTAAGGTGTTG	ATCTTACTCTGCTGCCTCTGATG	$(CTT)_5$	71–86
EDV-119	ACACCTTACGATCGACCTCCTAC	TCAACAAGTCTCAAAACCCAACT	$(GCA)_5$	95–125

Table S7. GenBank accession numbers of all newly generated cpDNA haplotypes (including outgroups) identified within the *Dysosma versipellis-pleiantha* complex.

Species	Haplotype	GenBank accession number		
		*trn*L–*trn*F	*trn*F–*ndh*J	*trn*S–*trn*fM
D. versipellis/*D. difformis*	H1	KT290738	KT290670	KT290806
D. versipellis	H2	KT290739	KT290671	KT290807
D. versipellis	H3	KT290740	KT290672	KT290808
D. versipellis	H4	KT290741	KT290673	KT290809
D. versipellis	H5	KT290742	KT290674	KT290810
D. versipellis	H6	KT290743	KT290675	KT290811
D. versipellis	H7	KT290744	KT290676	KT290812
D. versipellis	H8	KT290745	KT290677	KT290813
D. versipellis	H9	KT290746	KT290678	KT290814
D. versipellis	H10	KT290747	KT290679	KT290815
D. versipellis	H11	KT290748	KT290680	KT290816
D. versipellis	H12	KT290749	KT290681	KT290817
D. versipellis	H13	KT290750	KT290682	KT290818
D. versipellis	H14	KT290751	KT290683	KT290819
D. versipellis	H15	KT290752	KT290684	KT290820
D. versipellis	H16	KT290753	KT290685	KT290821
D. versipellis	H17	KT290754	KT290686	KT290822
D. versipellis	H18	KT290755	KT290687	KT290823
D. versipellis	H19	KT290756	KT290688	KT290824
D. versipellis	H20	KT290757	KT290689	KT290825
D. versipellis	H21	KT290758	KT290690	KT290826
D. versipellis	H22	KT290759	KT290691	KT290827
D. versipellis	H23	KT290760	KT290692	KT290828
D. versipellis	H24	KT290761	KT290693	KT290829
D. versipellis	H25	KT290762	KT290694	KT290830
D. versipellis	H26	KT290763	KT290695	KT290831
D. versipellis	H27	KT290764	KT290696	KT290832

D. versipellis	H28	KT290765	KT290697	KT290833
D. versipellis	H29	KT290766	KT290698	KT290834
D. versipellis	H30	KT290767	KT290699	KT290835
D. versipellis	H31	KT290768	KT290700	KT290836
D. versipellis	H32	KT290769	KT290701	KT290837
D. versipellis	H33	KT290770	KT290702	KT290838
D. versipellis	H34	KT290771	KT290703	KT290839
D. versipellis	H35	KT290772	KT290704	KT290840
D. versipellis	H36	KT290773	KT290705	KT290841
D. versipellis	H37	KT290774	KT290706	KT290842
D. versipellis	H38	KT290775	KT290707	KT290843
D. versipellis	H39	KT290776	KT290708	KT290844
D. versipellis	H40	KT290777	KT290709	KT290845
D. versipellis	H41	KT290778	KT290710	KT290846
D. versipellis	H42	KT290779	KT290711	KT290847
D. versipellis	H43	KT290780	KT290712	KT290848
D. versipellis	H44	KT290781	KT290713	KT290849
D. versipellis	H45	KT290782	KT290714	KT290850
D. versipellis	H46	KT290783	KT290715	KT290851
D. pleiantha	H47	KT290784	KT290716	KT290852
D. pleiantha	H48	KT290785	KT290717	KT290853
D. pleiantha	H49	KT290786	KT290718	KT290854
D. pleiantha	H50	KT290787	KT290719	KT290855
D. pleiantha	H51	KT290788	KT290720	KT290856
D. difformis	H52	KT290789	KT290721	KT290857
D. difformis	H53	KT290790	KT290722	KT290858
D. difformis	H54	KT290791	KT290723	KT290859
D. difformis/D. majoensis	H55	KT290792	KT290724	KT290860
D. versipellis	H56	KT290793	KT290725	KT290861
D. delavayi voucher 60		KT290794	KT290726	KT290862
D. delavayi voucher 61		KT290795	KT290727	KT290863
D. delavayi voucher 62		KT290796	KT290728	KT290864

D. delavayi voucher 63	KT290797	KT290729	KT290865
D. delavayi voucher 64	KT290798	KT290730	KT290866
D. delavayi voucher 65	KT290799	KT290731	KT290867
D. delavayi voucher 66	KT290800	KT290732	KT290868
D. aurantiocaulis voucher YN1	KT290801	KT290733	KT290869
D. aurantiocaulis voucher YN2	KT290802	KT290734	KT290870
D. tsayuensis	KT290803	KT290735	KT290871
Sinopodophyllum hexandrum	KT290736	KT290668	KT290804
Podophyllum peltatum	KT290737	KT290669	KT290805

References

Beaumont, M.A., Zhang, W. & Balding, D.J. Approximate Bayesian computation in population genetics. *Genetics* **162**, 2025-2035 (2002).

Chapuis, M. & Estoup, A. Microsatellite null alleles and estimation of population differentiation. *Mol. Biol. Evol.* **24**, 621-631 (2007).

Cornuet, J. et al. Inferring population history with DIY ABC: a user-friendly approach to approximate Bayesian computation. *Bioinformatics* **24**, 2713-2719 (2008).

Cornuet, J., Ravigné, V. & Estoup, A. Inference on population history and model checking using DNA sequence and microsatellite data with the software DIYABC (v1. 0). *BMC Bioinform.* **11**, 1 (2010).

Dempster, A.P., Laird, N.M. & Rubin, D.B. Maximum likelihood from incomplete data via the EM algorithm. *J. R. Stat. Soc. Series. B. Stat. Methodol.* **39**, 1-38 (1977).

Drummond, A.J. & Rambaut, A. BEAST: Bayesian evolutionary analysis by sampling trees. *BMC Evol. Biol.* **7**, 214 (2007).

Ellis, J.R. & Burke, J.M. EST-SSRs as a resource for population genetic analyses. *Heredity* **99**, 125-132 (2007).

Estoup, A., Jarne, P. & Cornuet, J.M. Homoplasy and mutation model at microsatellite loci and their consequences for population genetics analysis. *Mol. Ecol.* **11**, 1591-1604 (2002).

Evanno, G., Regnaut, S. & Goudet, J. Detecting the number of clusters of individuals using the software STRUCTURE: a simulation study. *Mol. Ecol.* **14**, 2611-2620 (2005).

Goudet, J. FSTAT, version 2.9.3.2. A program to estimate and test gene diversities and fixation indices. http://www2.unil.ch/popgen/siftwares/fstat.htm (2001).

Miller, M.A., Pfeiffer, W. & Schwartz, T. Creating the CIPRES Science Gateway for inference of large phylogenetic trees. Gateway Computing Environments Workshop (GCE), New Orleans, LA, USA (2010).

Posada, D. jModelTest: phylogenetic model averaging. *Mol. Biol. Evol.* **25**, 1253-1256 (2008).

Pritchard, J.K., Stephens, M. & Donnelly, P. Inference of population structure using multilocus genotype data. *Genetics* **155**, 945-959 (2000).

Qiu, Y., Zhou, X., Fu, C. & Chan, Y.G. A preliminary study of genetic variation in the endangered, Chinese endemic species *Dysosma versipellis* (Berberidaceae). *Bot. Bull. Acad. Sinica.* **46,** 61–69 (2005).

Rice, W.R. Analyzing tables of statistical tests. *Evolution* **43**, 223-225 (1989).

Rogers, A.R. & Harpending, H. Population growth makes waves in the distribution of pairwise genetic differences. *Mol. Biol. Evol.* **9**, 552-569 (1992).

Rogers, A.R. Genetic evidence for a Pleistocene population explosion. *Evolution* **49**, 608-615 (1995).

Ronquist, F. & Huelsenbeck, J.P. MrBayes 3: Bayesian phylogenetic inference under mixed models. *Bioinformatics* **19**, 1572-1574 (2003).

Rousset, F. Genepop'007: a complete re-implementation of the genepop software for Windows and Linux. *Mol. Ecol. Res.* **8**, 103-106 (2008).

Schneider, S. & Excoffier, L. Estimation of past demographic parameters from the distribution of pairwise differences when the mutation rates vary among sites: application to human mitochondrial DNA. *Genetics* **152**, 1079-1089 (1999).

Stamatakis, A., Hoover, P. & Rougemont, J. A rapid bootstrap algorithm for the RAxML web servers. *Syst. Biol.* **57**, 758-771 (2008).

Won, Y. & Hey, J. Divergence population genetics of chimpanzees. *Mol. Biol. Evol.* **22**, 297-307 (2005).
